# Loss of function of the ALS-associated NEK1 kinase disrupts microtubule homeostasis and nuclear import

**DOI:** 10.1126/sciadv.adi5548

**Published:** 2023-08-16

**Authors:** Jacob R. Mann, Elizabeth D. McKenna, Darilang Mawrie, Vasileios Papakis, Francesco Alessandrini, Eric N. Anderson, Ryan Mayers, Hannah E. Ball, Evan Kaspi, Katherine Lubinski, Desiree M. Baron, Liana Tellez, John E. Landers, Udai B. Pandey, Evangelos Kiskinis

**Affiliations:** ^1^ The Ken & Ruth Davee Department of Neurology, Feinberg School of Medicine, Northwestern University, Chicago, IL 60611, USA.; ^2^ Department of Pediatrics, Children’s Hospital of Pittsburgh, University of Pittsburgh School of Medicine, Pittsburgh, PA 15224, USA.; ^3^ Department of Neurology, University of Massachusetts Medical School, Worcester, MA 01605, USA.; ^4^ Simpson Querrey Institute, Northwestern University, Chicago, IL 60611, USA.; ^5^ Department of Neuroscience, Northwestern University Feinberg School of Medicine, Chicago, IL 60611, USA.

## Abstract

Loss-of-function variants in NIMA-related kinase 1 (NEK1) constitute a major genetic cause of amyotrophic lateral sclerosis (ALS), accounting for 2 to 3% of all cases. However, how *NEK1* mutations cause motor neuron (MN) dysfunction is unknown. Using mass spectrometry analyses for NEK1 interactors and NEK1-dependent expression changes, we find functional enrichment for proteins involved in the microtubule cytoskeleton and nucleocytoplasmic transport. We show that α-tubulin and importin-β1, two key proteins involved in these processes, are phosphorylated by NEK1 in vitro. NEK1 is essential for motor control and survival in *Drosophila* models in vivo, while using several induced pluripotent stem cell (iPSC)–MN models, including NEK1 knockdown, kinase inhibition, and a patient mutation, we find evidence for disruptions in microtubule homeostasis and nuclear import. Notably, stabilizing microtubules with two distinct classes of drugs restored NEK1-dependent deficits in both pathways. The capacity of NEK1 to modulate these processes that are critically involved in ALS pathophysiology renders this kinase a formidable therapeutic candidate.

## Teaser

NEK1 is an ALS-associated multifaceted kinase that modulates microtubule homeostasis and nuclear import in human motor neurons.

## INTRODUCTION

Amyotrophic lateral sclerosis (ALS) is a devastating neurodegenerative disease characterized by the dysfunction and degeneration of upper and lower motor neurons (MNs) found in the brain and spinal cord, respectively (*1*). Clinically, the loss of MNs manifests as progressive loss of voluntary muscle movement, which leads to paralysis and eventual death (*1*). The known genetic etiology of ALS, which accounts for up to 65% of all familial and 10% of all sporadic cases, is complex, with more than 30 disease-causing genes identified to date (*2*–*5*). The genes encode proteins that exhibit a high degree of functional heterogeneity, spanning such cellular pathways as RNA metabolism, proteostasis, cytoskeletal homeostasis, and intracellular trafficking (*2*, *3*). An array of recent genetic studies based on whole-exome sequencing has identified that ALS patients exhibit a significant enrichment of predicted loss-of-function heterozygous variants in the gene encoding *NIMA-related kinase 1* (*NEK1*), which accounts for 2 to 3% of both familial and sporadic disease (*6*–*14*). The mechanisms by which NEK1 haploinsufficiency causes MN dysfunction and eventual degeneration remain unknown.

**Fig. 1.**
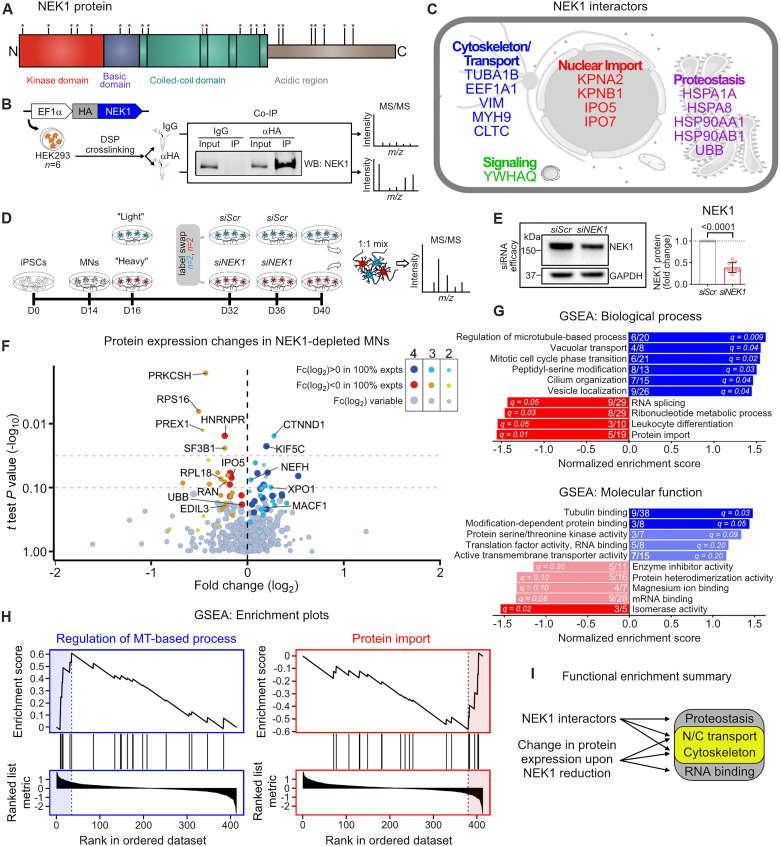
Proteomics analysis of NEK1 interactors and expression changes converges on MT homeostasis and N/C transport. (**A**) NEK1 protein domain structure. Observed case-control *NEK1* nonsense variants found in ALS cohorts are represented with asterisks (*6*–*12*). The mutation used in this investigation is marked in red (R540X). (**B**) Experimental schematic used to identify NEK1 interactors by LC-MS/MS. (**C**) Identified NEK1 interactors grouped according to cellular function. (**D**) Experimental schematic used to identify differential protein expression upon NEK1 reduction in iPSC-MNs. (**E**) Left: Representative WB for NEK1 in *siScr*- and *siNEK1*-treated MN cultures. GAPDH was used as a loading control. Right: Fold change in NEK1 levels following *siNEK1* treatment. Circles = individual samples; dotted line = mean NEK1 levels in *siScr*-treated MNs. *n* = 10 independent differentiations. (**F**) Volcano plot representing the fold change in expression level in *siNEK1*-treated versus *siScr*-treated MNs on the *x* axis and the statistical significance of the change (log_10_ of *P* value) on the *y* axis. Each circle represents a unique protein identified. Purple/blue circles = up-regulated proteins; red/orange circles = down-regulated proteins. Circle size and shade reflect the number of experiments where the protein was identified. Dashed red lines = *P* values of 0.05 and 0.1. (**G**) Enriched terms from ranked proteins shown in (F) using Gene Set Enrichment Analysis (GSEA) for biological process (top) and molecular function (bottom). Bars represent normalized enrichment scores for each term. Blue bars = up-regulated proteins; red bars = down-regulated proteins. Numbers within bars represent the number of up-regulated/down-regulated proteins in our dataset (numerator) out of all proteins denoted in each category (denominator). *Q* values are shown within individual bars. (**H**) Representative enrichment plots for the top enriched terms for up-regulated proteins (left) and down-regulated proteins (right) corresponding to the GSEA shown in (G). (**I**) Schematic representing the functional pathways highlighted based on LC-MS/MS experiments.

NEK1 belongs to a diverse family of highly conserved serine/threonine kinases involved in cell cycle control, ciliogenesis, and the DNA damage response (DDR) (*15*–*18*). In dividing cells under basal conditions, NEK1 is primarily localized to the centrosome at the base of the primary cilium, where it stabilizes the microtubule (MT) axial filament of cilia (*19*, *20*). In line with a prominent role of NEK1 in ciliogenesis, homozygous *NEK1* mutations cause a perinatally fatal ciliopathy known as short rib-polydactyly syndrome type II (SRPS II) (*20*, *21*). The ALS-associated nonsense genetic variants, which are predominantly heterozygous, are scattered across the entirety of the protein including within the enzymatic domain, the coiled-coil domain that mediates protein-protein interactions, and the C-terminal acidic region (Fig. 1A) (*6*–*12*).

Although there are very few well-characterized NEK1 target phosphorylation sites, NEK1 interactors functionally converge on the MT cytoskeleton (*6*). MTs are polar cytoskeletal polymers composed of α-tubulin and β-tubulin heterodimers. They support cytoarchitecture and form tracks for organelle, protein, and RNA cargo transport (*22*). MTs can be dynamic, undergoing constant growth and shrinkage, or stable, where they are characterized by resistance to depolymerization (*23*). Neurons contain a highly distinct MT cytoskeleton characterized by diverse genetic and chemical tubulin composition, an abundance of stable MTs, and acentrosomal MT nucleation (*23*–*26*). NEK1 also functions in the DDR pathway in dividing cells where, upon a DNA insult, it translocates to the nucleus and colocalizes with the canonical DNA damage marker γ-H2AX (*27*). The relevance of these NEK1 mechanisms in human neurons and, specifically, their relative contribution to ALS pathogenesis remain largely undetermined. There has only been one cell-based study investigating *NEK1*-related ALS, which showed that neurons derived from a single ALS patient induced pluripotent stem cell (iPSC) line with a heterozygous mutation expressed 50% less protein and exhibited higher levels of DNA damage relative to unrelated healthy controls (*28*).

Here, we sought to determine how NEK1 haploinsufficiency causes MN dysfunction. We show that NEK1 interactors are enriched for function in the MT cytoskeleton and nucleocytoplasmic (N/C) transport, and that reduction of NEK1 levels results in differential expression of proteins involved in these pathways. Using several iPSC-derived MN models, we demonstrate that NEK1 loss of function disrupts TUBA1B-dependent MT homeostasis, KPNB1 localization, and nuclear import. We also find that NEK1 is essential for motor control and survival in *Drosophila* models in vivo. Our findings highlight NEK1 as a pleiotropic kinase with regulatory roles in two critical pathways for MN function that have previously been implicated in ALS pathogenesis.

## RESULTS

### Proteomics analysis of NEK1 interactors and expression changes converges on microtubule homeostasis and nucleocytoplasmic transport

Protein phosphorylation by kinases is the most common posttranslational modification (*29*, *30*). It modulates protein activity and localization, as well as protein-protein interactions, and indirectly controls the expression level of target proteins (*29*, *30*). To begin interrogating how *NEK1* haploinsufficiency may cause neuronal dysfunction, we sought to identify NEK1 interactors as potential phosphorylation targets using affinity purification mass spectrometry (AP-MS). We expressed hemagglutinin (HA)–tagged NEK1 protein in human embryonic kidney (HEK) 293FT cells and performed immunoprecipitation coupled to liquid chromatography–tandem mass spectrometry (LC-MS/MS) across multiple experiments (Fig. 1B). We used the reversible crosslinker dithiobis(succinimidyl propionate) (DSP) to stabilize transient signaling interactions, a strategy that has been previously used to identify interactors of other kinases (Fig. 1B and fig. S1A) (*31*, *32*). As expected, DSP treatment (1 mM, 30 min) induced the formation of high–molecular weight complexes of both endogenous NEK1 and exogenous HA-NEK1 (fig. S1A), but did not interfere with HA-NEK1 immunoprecipitation, and was reversible following exposure of samples to reducing conditions before LC-MS/MS (fig. S1B).

LC-MS/MS analysis revealed 15 unique NEK1 interactors that clustered into three broad functional groups: cytoskeletal homeostasis, N/C transport, and proteostasis (Fig. 1C). All three of these pathways have been previously implicated in ALS pathogenesis through both genetic analysis and observations from disease models and postmortem patient tissue (*5*, *33*, *34*). Cytoskeleton-associated NEK1 interactors include α-tubulin (TUBA1B), which is a primary structural component of MTs, elongation factor 1-α (EF1a), which can sever and stabilize MTs, the MT-modulating coat protein clathrin heavy chain 1 (CLTC), intermediate filament vimentin (VIM), and actin motor myosin 9 (MYH9). NEK1 also interacted with four distinct nuclear import receptors including importin-α1 (KPNA2), importin-β1 (KPNB1), importin-5 (IPO5), and importin-7 (IPO7), which are collectively responsible for transporting a diverse class of proteins into the nucleus (*35*). The last functional group includes proteins involved in protein homeostasis such as heat shock proteins (HSPA1A, HSPA8, HSP90A1, and HSP90AB1) and ubiquitin (UBB). To validate these findings, we selected nine NEK1-interacting proteins spanning each functional category and two negative control proteins not identified to interact with NEK1 and performed co-immunoprecipitation coupled to Western blot (WB) analysis. We found that all nine of the identified NEK1-interacting proteins, but neither of the negative control proteins (CLIMP63 and Histone H3) that we assessed, coprecipitated with HA-NEK1, validating the LC-MS–based strategy (fig. S1C).

We next sought to identify changes in protein expression levels upon NEK1 reduction in human MNs. We focused on decreasing NEK1 levels to simulate haploinsufficiency that has been reported to occur in *NEK1*-ALS patients (*6*–*8*, *28*). Using previously established protocols (*36*), we differentiated spinal MNs from a control iPSC line [line 18a (*37*); table S1], allowed them to mature for 40 days in vitro (fig. S1, D and E), and sequentially treated twice with a small interfering RNA (siRNA) targeting *NEK1* or a scrambled control (*siNEK1* or *siScr*) (Fig. 1D). This approach caused a 50 to 60% reduction in full-length NEK1 protein levels (*P* < 0.0001) (Fig. 1E). To accurately quantitate any proteome-wide alterations, we used stable isotope labeling with amino acids in cell culture (SILAC). We pulse-labeled MN cultures for a total of 24 days in vitro (days 16 to 40), processed whole-cell extracts, mixed the two conditions at a 1:1 ratio (*siScr* and *siNEK1*), and performed MS analysis (Fig. 1D). To control for potential condition-specific bias, we performed label swapping, where we SILAC-pulsed *siNEK1*-treated MNs in two experiments, and SILAC-pulsed *siScr*-treated MNs in the other two experiments. The quantification of labeling efficiency showed that across all four experiments we successfully labeled 80% of all peptides at 80%, and at least 65% of all peptides at 100% efficiency (fig. S1F). While there was variability in the identities of the proteins detected across differentiations, we identified 201 common proteins in all four experiments (fig. S1G). To identify proteins of interest, we initially applied a stringent *P* value of <0.05 that yielded only a handful of differentially expressed proteins between *siScr*- and *siNEK1*-treated samples (Fig. 1F). Given the complex experimental design (see Materials and Methods), we additionally considered the consistency of change across experiments, i.e., up-regulation or down-regulation upon NEK1 reduction for a given protein independent of label swapping, in combination with a lower threshold in statistical significance (*P* < 0.1) (Fig. 1F and data S1). This analysis yielded 28 differentially expressed proteins in *siNEK1*-treated MNs. Among these, we identified and validated down-regulation of IPO5 in NEK1-depleted MNs (fig. S1H), a key nuclear import protein and NEK1 interactor identified in the AP-MS/MS experiments described above (Fig. 1, C and F).

To determine whether any unifying functional features existed among the group of differentially expressed proteins, we first performed Gene Set Enrichment Analysis (GSEA) (Fig. 1, G and H). The highest enrichment scores for up-regulated proteins included the terms “regulation of microtubule-based process” for biological process and “tubulin binding” for molecular function (Fig. 1, G and H). Enriched terms for down-regulated proteins included “protein import” and “RNA splicing” for biological process, as well as “mRNA binding” for molecular function (Fig. 1, G and H). We also observed enrichment of terms previously associated with NEK1 function (*38*), such as “cilium organization,” “mitotic cell phase transition,” and “protein serine/threonine kinase activity,” increasing our confidence in these experiments. To complement the GSEA, we also performed gene ontology (GO) analysis using the Database for Annotation, Visualization and Integrated Discovery (DAVID) and interaction network analysis using the Search Tool for the Retrieval of Interacting Genes/Proteins (STRING). For up-regulated proteins, “MT binding” was by far the most enriched molecular function pathway (fig. S1I). STRING supported these findings, with the highest network enrichment in GO terms for molecular function being “dynein complex binding,” “MT binding,” and “motor activity” (fig. S1I). For down-regulated proteins, GO analysis revealed an enrichment for “RNA binding” and “nuclear pore” (fig. S1J). Together, our two independent and unbiased proteomics-based experiments suggest that NEK1 loss of function may directly affect the MT cytoskeleton and N/C transport (Fig. 1I).

### NEK1 interacts with TUBA1B and KPNB1 in MNs and phosphorylates them in vitro

**Fig. 2.**
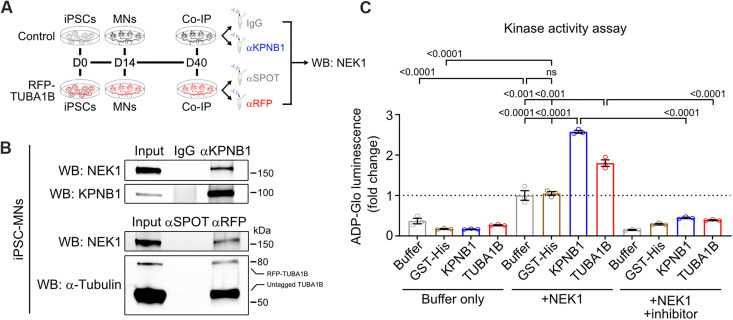
KPNB1 and TUBA1B interact with endogenous NEK1 in MNs and can undergo NEK1-mediated phosphorylation in vitro. (**A**) Experimental schematic to confirm interaction between endogenous NEK1 and KPNB1/TUBA1B. Antibodies targeting KPNB1 or RFP were used in coimmunoprecipitation (Co-IP) from day 40 MN lysates differentiated from control (top) or endogenously tagged RFP-TUBA1B (bottom) iPSC lines. (**B**) Top: Representative WB using iPSC-MN lysates immunoprecipitated with IgG (control) or αKPNB1 and probed for NEK1. Bottom: Representative WB using RFP-TUBA1B iPSC-MN lysates immunoprecipitated with αSPOT beads (control) or αRFP beads and probed for NEK1. (**C**) Bar plots showing in vitro phosphorylation of recombinant KPNB1/TUBA1B, but not the control GST-His protein, by NEK1 as measured by ATP/ADP conversion. Buffer only reactions (left) consist of kinase reaction buffer with 100 ng of indicated substrate. +NEK1 reactions (middle) consist of kinase reaction buffer with 100 ng of indicated substrate and 10 ng of active NEK1. +NEK1 +inhibitor reactions (right) consist of kinase reaction buffer with 100 ng of indicated substrate, 10 ng of active NEK1, and 1 μM staurosporine. All conditions include 50 μM ATP. *n* = 3 reactions; bars represent mean ± SD, adjusted *P* values noted above comparisons.

Given the convergence of the MS-based data upon MT homeostasis and N/C transport, we next selected α-tubulin (TUBA1B) and importin-β1 (KPNB1) as two key proteins playing essential roles in these two pathways, respectively, and investigated whether they may be directly regulated by NEK1. We first sought to confirm the interaction of TUBA1B and KPNB1 with endogenous NEK1 in human MNs (Fig. 2A). We differentiated spinal MNs from a healthy control iPSC line, and one line with an RFP tag incorporated within the *TUBA1B* gene (line 18a/RFP-TUBA1B; table S1). We pulled down endogenous KPNB1 and TUBA1B and found substantial coprecipitation of native NEK1 in both cases (Fig. 2, A and B). We did not observe coprecipitation of negative control proteins with KPNB1 (CLIMP63 and EB3) or TUBA1B (TDP-43) (fig. S2, A and B), validating the specificity of NEK1 interactions observed in these experiments.

We next used an in vitro kinase assay that detects adenosine triphosphate (ATP) to adenosine diphosphate (ADP) conversion upon protein phosphorylation and has been shown to measure NEK kinase activity (*39*), to determine whether TUBA1B and KPNB1 are phosphorylation substrates of NEK1. We found that while NEK1 undergoes a basal level of autophosphorylation (*40*), addition of recombinant TUBA1B or KPNB1, but not the control glutathione *S*-transferase (GST)–His protein, significantly increased ATP-ADP conversion in these reactions, demonstrating NEK1 phosphorylation of TUBA1B/KPNB1 in this recombinant system (Fig. 2C). This effect was blocked by the addition of the kinase inhibitor staurosporine, demonstrating that NEK1 kinase activity was responsible for increased ATP-ADP conversion. Notably, an in silico analysis of the amino acid sequences of TUBA1B and KPNB1 using PhosphoSVM and GPS 5.0 prediction software (*41*–*43*) and a database of the NEK enzyme kinome (*44*) revealed several candidate serine/threonine residues that are likely NEK1 phosphorylation sites (table S2). Collectively, these data demonstrate that NEK1 associates physiologically with TUBA1B and KPNB1 in human MNs, and can phosphorylate these proteins in vitro, suggesting that it may regulate their critical function.

### Reduction of NEK1 levels in MNs disrupts MT homeostasis

**Fig. 3.**
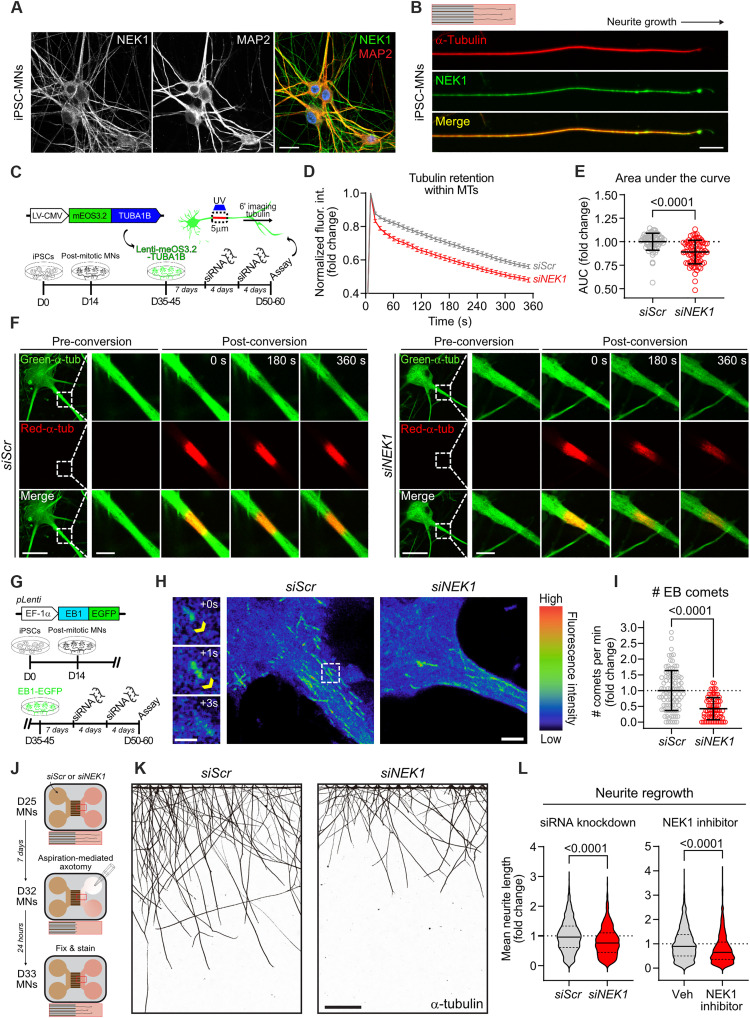
Reduction of NEK1 levels in MNs disrupts MT homeostasis. (**A**) Representative image of day 50 control MNs immunostained for NEK1 (green), MAP2 (red), and DAPI (blue). Scale bar, 20 μm. (**B**) Representative image of a day 32 control MN neurite cultured in a microfluidic device and immunostained for NEK1 (green) and α-tubulin (red). Scale bar, 20 μm. (**C**) Experimental schematic used to measure tubulin mobility in MN MTs. (**D**) Representative images showing tubulin mobility assay in *siScr-* and *siNEK1*-treated MNs. Dashed lines = inset region magnified in the right images. Scale bars, 25 μm and 5 μm (inset). (**E**) Persistence of photoconverted mEos3.2-TUBA1B fluorescence over time. *n* = 3 independent differentiations; data represented as mean ± SEM. (**F**) Fold change in the area under curve (AUC) from line plots shown in (C). Circles = individual cells; dotted line = mean AUC in control MNs. *n* = 4 independent differentiations. (**G**) Experimental schematic used to measure MT polymerization in proximal MN neurites. (**H**) Left: Representative time-lapse images of EB comets. Yellow arrow = EB comet. Right: Representative maximum intensity in time projections of total MT trajectories from EB1-GFP live imaging of MNs. Dashed lines = inset region magnified in the left images. Scale bars, 10 μm and 1 μm (inset). (**I**) Number of EB comets per minute in *siScr*- and *siNEK1*-treated MNs. Circles = individual cells. *n* = 3 independent differentiations. (**J**) Experimental schematic used to measure neurite regeneration following axotomy of MNs. (**K**) Representative images of *siScr-* and *siNEK1-*treated MN neurites 24 hours after axotomy immunostained for α-tubulin. Scale bar, 200 μm. (**L**) Fold change of individual neurite regrowth length of *siScr-* or *siNEK1-*treated (left) and vehicle- or 10 μM NEK1 inhibitor–treated (right) MNs. Data represented as median (bold) and quartiles (dashed) within plots. *n* = 3 to 4 independent differentiations. All individual *P* values are shown above comparisons. Data are represented as mean ± SD unless otherwise noted.

NEK1 exhibits diffuse localization throughout the cell body in differentiated MNs that is very pronounced within neuronal processes, and strongly colocalizes with TUBA1B and microtubule-associated protein (MAP2) (Fig. 3, A and B). Given the strong colocalization and interaction of NEK1 with TUBA1B in MNs and the ability of NEK1 to phosphorylate it, we next asked whether NEK1 can modulate TUBA1B homeostasis. We used TUBA1B fused to mEos3.2, a photoconvertible fluorescent protein, whose emission spectrum changes from green (516 nm) to red (581 nm) upon ultraviolet light stimulation (Fig. 3C) (*45*). Tubulin molecules incorporated into stable MTs are trapped and immobile, whereas soluble tubulin and tubulin incorporated into dynamic MTs may rapidly diffuse through the cytosol. We transduced MNs with mEos3.2-TUBA1B and established that before stimulation, the fluorescence signal was diffuse and filamentous (fig. S3A). We photoconverted a 5 μm region of proximal neurite and quantified the fluorescence intensity of red–α-tubulin and green–α-tubulin over 5 min. To account for the total available pool of α-tubulin within the photoconverted region across time, which can be highly variable across genotypes and between neurons, we normalized photoconverted red–α-tubulin to green–α-tubulin intensity values. To ensure that our assay was an effective measure of TUBA1B retention, we treated MNs with the MT-stabilizing drug paclitaxel (PTX; 50 nM) or the MT-depolymerizing drug colchicine (Colch; 1 μM). As expected, PTX caused a significant persistence of red/green fluorescence over time in both *siScr*- (*P* < 0.0001) and *siNEK1*-treated (*P* < 0.001) MNs, while Colch elicited the converse effect (*P* < 0.0001) (fig. S3, A to D). Critically, MNs with reduced NEK1 expression following *siNEK1* treatment exhibited a significant reduction in TUBA1B retention within the stimulated region, compared to *siScr*-treated controls (*P* < 0.0001) (Fig. 3, D to F). We observed similar results when we performed this assay in MNs differentiated from a second control iPSC line (line CS002, see Materials and Methods), demonstrating that the effects of NEK1 depletion on TUBA1B homeostasis in proximal MN neurites were independent of genetic background (*P* < 0.01) (fig. S3, E and F).

We next interrogated the effect of NEK1 reduction on the pool of dynamic MTs by using an established EB1-GFP (green fluorescent protein) reporter assay that provides a sensitive measurement of active MT polymerization (*46*). MTs polymerize unidirectionally, with tubulin dimers incorporated into the growing plus end, which is bound by the end-binding protein EB1 (*24*). We differentiated control MNs, infected them with a lentivirus expressing EB1-GFP, and used live imaging in proximal MN neurites to measure the number of polymerization events occurring per minute, commonly referred to as “EB comets” (Fig. 3G) (*24*). Critically, treatment with nocodazole (NDZ; 10 μM), which causes MT depolymerization at high concentrations, led to a marked reduction in the number of EB1 comets, validating this approach (*P* < 0.0001) (fig. S3, G and H). MNs treated with *siNEK1* exhibited a significant reduction in the number of detected EB comets when compared to MNs treated with *siScr* (*P* < 0.0001), demonstrating impaired MT polymerization (Fig. 3, H and I). To assess the relative specificity of the effects of NEK1 loss of function on MTs versus other components of the cytoskeleton, we analyzed the levels of filamentous actin (F-actin) by immunocytochemistry (ICC). Reduction of NEK1 in MNs did not cause a significant change in F-actin levels, in either the somatic or proximal dendritic regions of *siNEK1*-treated MNs (*P* = 0.6227 and *P* = 0.7329, respectively) (fig. S3, I and J). However, more in-depth analysis would be required to eliminate any NEK1-dependent effects on the general actin network or more subtle actin-based structures and processes.

Given the effects of NEK1 reduction on TUBA1B retention and MT polymerization we next interrogated axonal regeneration as a neuronal process that relies heavily on proper coordination of MT dynamics (*47*) and known to be compromised in ALS models (*48*–*51*). MNs were cultured for 11 days in microfluidic devices to isolate neurites from cell bodies and treated with control or NEK1 siRNAs for 7 days (Fig. 3J). Acute axotomy was then performed by constant aspiration/reperfusion within the axonal compartment. Following a 24-hour regeneration period, MNs were fixed and immunolabeled with TUBA1B (Fig. 3K). Automated image analysis of individual neurite length demonstrated a significant decrease in axonal outgrowth in *siNEK1*-treated compared to *siScr*-treated MNs (*P* < 0.0001) (Fig. 3, K and L), suggesting that NEK1 loss of function may impair neurite repair following injury. Critically, these defects were dependent on the enzymatic activity of the kinase, as we could recapitulate them using a small-molecule NEK1-specific inhibitor (NEK1i) (*P* < 0.0001) (Fig. 3K) (*52*). Together, our findings demonstrate that NEK1 loss of function disrupts TUBA1B homeostasis within neuronal processes and negatively affects downstream processes like neurite regeneration.

### Reduction of NEK1 levels in MNs perturbs N/C transport

**Fig. 4.**
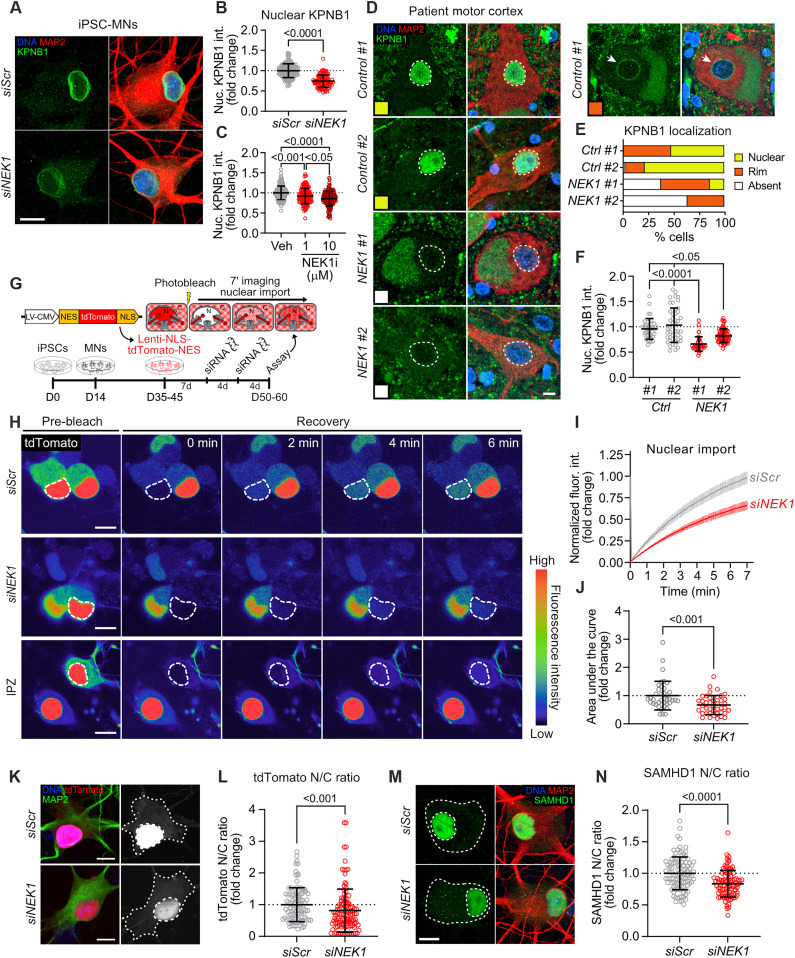
Reduction of NEK1 levels in MNs perturbs N/C transport. (**A**) Representative images of *siScr*- and *siNEK1*-treated MNs immunolabeled for KPNB1 (green), MAP2 (red), and DAPI (blue). (**B** and **C**) Fold change of nuclear KPNB1 fluorescence intensity in *siScr*- and *siNEK1*-treated (B) or vehicle- and NEK1 inhibitor–treated (C) MNs. *n* = 3 independent differentiations. (**D**) Representative images of two control and two *NEK1*-ALS motor cortex sections immunolabeled for KPNB1 (green), MAP2 (red), and DAPI (blue). Colored box = KPNB1 localization pattern (nuclear = yellow, nuclear rim = orange, absent = white). (**E**) Percentage of MNs from each patient with the indicated KPNB1 localization. *n* = 35 to 61 neurons. (**F**) Fold change of nuclear KPNB1 fluorescence intensity in control and *NEK1*-ALS patient motor cortex MNs. *n* = 2 patients per disease condition. (**G**) Experimental schematic used to measure nuclear import dynamics in MNs by FRAP imaging of an NES-tdTomato-NLS reporter. (**H**) Representative images showing FRAP assay in *siScr-* and *siNEK1*-treated MNs. (**I**) Percent fluorescence recovery in the nucleus over time. *n* = 3 independent differentiations; data are represented as mean ± SEM. (**J**) Fold change in the AUC from line plots shown in (I). (**K**) Representative images of *siScr*- and *siNEK1*-treated MNs expressing the NES-tdTomato-NLS reporter (red) immunolabeled for MAP2 (green) and DAPI (blue). (**L**) Fold change in the N/C ratio of the NES-tdTomato-NLS reporter. *n* = 3 independent differentiations. (**M**) Representative images of *siScr*- and *siNEK1*-treated MNs immunolabeled for SAMHD1 (green), MAP2 (red), and DAPI (blue). (**N**) Fold change in the N/C ratio of SAMHD1. *n* = 3 independent differentiations. All individual *P* values are shown above comparisons. Data are represented as mean ± SD unless otherwise noted. For all plots, circles = individual neurons; dotted line = mean of control neurons. For all images, dashed lines = nucleus; dotted lines = soma. All scale bars = 10 μm.

Our proteomics experiments highlighted N/C transport as another pathway likely targeted for NEK1-dependent regulation (Fig. 1 and fig. S1). This pathway mediates the transport of molecules between the nucleus and the cytoplasm. Cargos that shuttle in and out of the nucleus typically carry a nuclear localization sequence (NLS) that can be recognized by a nuclear import receptor, and a nuclear export sequence (NES) that can be recognized by a nuclear export receptor (*35*). Distinct nuclear transport receptors mediate the transport of specific cargos through the nuclear pore complex (NPC) embedded within the nuclear envelope. KPNB1 is one such nuclear transport receptor that we identified as a physiological NEK1 interactor in iPSC-derived MNs and a NEK1 phosphorylation target in vitro. We found that MNs differentiated from two distinct healthy control iPSC lines (18a and CS002; table S1) exhibited a significant reduction in KPNB1 intensity levels within the nucleus and along the nuclear envelope following treatment with *siNEK1* relative to *siScr* controls (Fig. 4, A and B, and fig. S4, A and B). These defects were dependent on NEK1 kinase activity, as we could recapitulate them by treating MNs with the small-molecule NEK1 inhibitor (Fig. 4C and fig. S4C). To determine whether similar alterations in KPNB1 levels are present in human ALS patients, we next performed immunohistochemistry in motor cortex sections of two control and two *NEK1*-ALS patients harboring missense *NEK1* mutations (Fig. 4, D to F; fig. S4D; and table S3). KPNB1 distribution was markedly variable in postmortem control tissue, with some neurons showing diffuse nuclear staining (53 to 78%, yellow), and others an enrichment along the nuclear envelope (20 to 47%, orange) (Fig. 4, D and E). This pattern was also observed in a minority of *NEK1*-ALS patient neurons (37 to 48%). However, a substantial proportion of *NEK1*-ALS patient neurons displayed a near-complete absence of KPNB1 signal in the nucleus and around the nuclear envelope (37 to 63%, white), which was not observed in control patient neurons (0 to 2%). *NEK1*-ALS patient neurons also exhibited a significant reduction in mean KPNB1 intensity within the nucleus relative to controls (Fig. 4F), in accordance with findings in iPSC-derived MNs treated with *siNEK1*. Last, while we observed a significant correlation between nuclear KPNB1 and nuclear TDP-43 signal in postmortem motor cortex control neurons (fig. S4D), there was no such correlation in NEK1 patients.

KPNB1 and KPNA2, both of which we identified as NEK1 interactors (Fig. 1C), cooperate for the nuclear import of proteins harboring a classical NLS (cNLS) (*53*). To assess the effects of NEK1 loss of function on active KPNB1-mediated nuclear import, we used a fluorescent tdTomato reporter encoding a cNLS and an NES that can shuttle between the nucleus and the cytoplasm, but predominantly localizes to the nucleus (Fig. 4G). We delivered the reporter to differentiated MNs via lentiviral transduction, treated the cultures with *siNEK1* or *siScr*, and performed fluorescent recovery after photobleaching (FRAP) imaging by live-cell confocal microscopy on neuronal nuclei to measure nuclear import rates (Fig. 4G) (*54*). We first validated the assay by treating MN cultures with importazole (IPZ; 20 μM), which binds to KPNB1 to block nuclear import (fig. S4, E and F) (*55*). We also measured tdTomato fluorescence intensity before FRAP imaging to ensure that there were no differences in reporter expression levels resulting from siRNA treatments (fig. S4G). FRAP analysis revealed a significant reduction in the rate of nuclear import in *siNEK1*-treated MNs relative to cultures treated with *siScr* (*P* < 0.001) (Fig. 4, H to J). In parallel to live-cell imaging, we performed ICC on MNs expressing the same cNLS/NES reporter at a fixed time point following treatment with *siNEK1* or *siScr* and measured the N/C ratio of tdTomato (*56*). This analysis showed that NEK1 knockdown led to a significant shift in the reporter signal from the nucleus to the cytoplasm, in accordance with the findings of the FRAP assay described above (*P* < 0.05) (Fig. 4, K and L). Last, we investigated whether NEK1 loss of function affects the import of endogenous KPNB1 cargoes. We selected the cNLS-bearing SAMHD1 as a representative cargo protein as it has been previously shown to be imported into the nucleus by KPNB1/KPNA2 (*57*). We found that *siNEK1*-treated MNs exhibited a significantly reduced N/C ratio of SAMHD1 compared to controls (Fig. 4, M and N). Together, these data suggest that NEK1 can modulate KPNB1 localization and function, and that NEK1 loss of function compromises KPNB1/KPNA2-mediated nuclear import.

### MT stabilization improves nuclear import in NEK1-depleted neurons

**Fig. 5.**
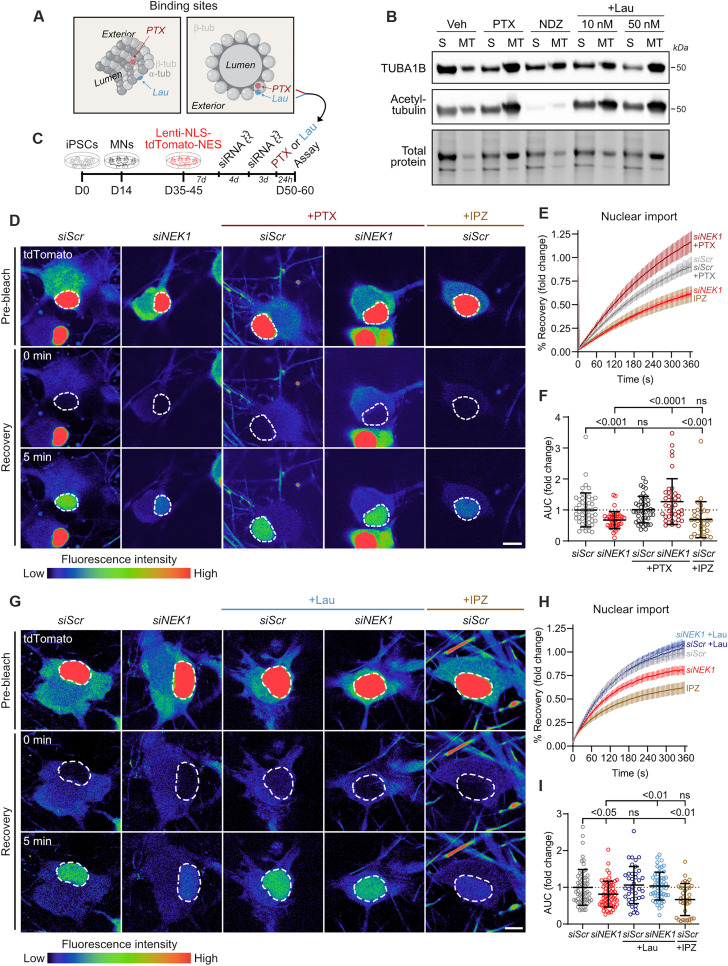
MT stabilization restores nuclear import deficits in NEK1-depleted MNs. (**A**) Schematic displaying the binding sites of the MT-stabilizing drugs paclitaxel (PTX) and laulimalide (Lau) on β-tubulin within MTs. (**B**) Representative WB following MT fractionation of vehicle-, PTX-, NDZ-, and Lau-treated iPSC-MN cultures showing soluble:polymerized α-tubulin and acetylated α-tubulin in the MT fraction. (**C**) Experimental schematic used to measure nuclear import levels in control or NEK1-depleted MNs. (**D**) Representative live images showing FRAP assay in *siScr*- and *siNEK1*-treated MNs following vehicle or PTX treatment. IPZ-treated MNs are included as a control. Dashed lines = photobleached region (nucleus). Scale bars, 10 μm. (**E**) Percent recovery of reporter fluorescence intensity in the photobleached region of the nucleus over time. *n* = 4 independent differentiations; data are represented as mean ± SEM. (**F**) Fold change in the AUC from the line plot shown in (E) in *siScr*- and *siNEK1*-treated MNs following vehicle, PTX, or IPZ treatment. Circles = individual cells; dotted line = mean AUC in control MNs. *n* = 4 independent differentiations. (**G**) Representative live confocal images showing FRAP assay in *siScr*- and *siNEK1*-treated MNs following vehicle or Lau treatment. IPZ-treated MNs are included as a control. Dashed lines mark the photobleached region (nucleus). Scale bars, 10 μm. (**H**) Percent recovery of reporter fluorescence intensity in the photobleached region of the nucleus over time. *n* = 3 independent differentiations; data are represented as mean ± SEM. (**I**) Fold change in the AUC from the line plot shown in (H) in *siScr*- and *siNEK1*-treated MNs following vehicle, Lau, or IPZ treatment. Circles = individual cells; dotted line = mean AUC in control MNs. *n* = 3 independent differentiations. All individual *P* values are shown above comparisons. Data are represented as mean ± SD unless otherwise noted.

Given the structural and functional link between the cytoskeleton and the N/C pathway (*58*–*60*), we next asked whether increasing the abundance of stable MTs might modulate N/C transport in the context of NEK1 loss of function. To test this hypothesis, we used two MT-stabilizing drugs with distinct β-tubulin binding sites: (i) PTX, which binds at the taxane site within the MT lumen, and (ii) laulimalide (Lau), which binds at the Lau/peloruside site on the exterior of MTs (Fig. 5A) (*61*). While the mechanisms by which these two molecules promote MT stabilization are not definitively established, studies have suggested distinct yet slightly overlapping modes of action (*61*, *62*). Findings demonstrating the synergistic effect of drugs acting on these two sites further support this notion (*63*). To confirm the MT stabilization activity of PTX and Lau in iPSC-MNs, we performed biochemical MT fractionation in day 40 MNs following a 24-hour treatment with either drug (0 to 50 nM, 4 hours) or the MT-destabilizing drug NDZ (10 μM, 4 hours) as a negative control (Fig. 5B and fig. S5, A and B). WB analysis of polymerized:soluble TUBA1B (fig. S5A) and polymerized acetylated α-tubulin, which is a marker of stable MTs, within the MT fraction (fig. S5B) confirmed similar MT stabilization levels at 50 nM concentrations of both PTX and Lau.

To assess the ability of these drugs to modulate N/C transport, we measured nuclear import in live MNs using the tdTomato-cNLS/NES reporter following NEK1 reduction and a 24-hour treatment with 50 nM of either PTX or Lau (Fig. 5C). We found that while knockdown of NEK1 reduced nuclear import (*siScr* versus *siNEK1*, *P* < 0.001), PTX caused a substantial increase, to levels equal to those observed in untreated siScr-MNs (*siNEK1* versus *siNEK1* + PTX, *P* < 0.0001) (Fig. 5, D to F). Critically, this treatment did not affect expression of the tdTomato-cNLS/NES reporter as measured by baseline fluorescence intensity (fig. S5C). While PTX treatment caused a significant increase in NEK1 protein levels in *siScr* control MNs, no such effect was observed in the context of the NEK1 knockdown (fig. S5, D and E), discounting the possibility that the PTX rescue was driven by increasing NEK1 levels. In additional experiments, we observed a similar rescue of NEK1-dependent nuclear import deficits (*siScr* versus *siNEK1*, *P* < 0.05) following treatment with Lau (*siNEK1* versus *siNEK1* + Lau, *P* < 0.01) (Fig. 5, G to I), further substantiating the functional interaction between MT stability and nuclear import. These treatments again did not appear to affect tdTomato-cNLS/NES expression (fig. S5F), nor did Lau treatment cause any changes in NEK1 protein expression in either *siScr*- or *siNEK1*-treated MNs (fig. S5, G and H). Together, these experiments illustrate a mechanistic connection between MT stability and N/C trafficking and showcase the ability of MT stabilization to rescue N/C import in NEK1-deficient MNs.

### ALS patient mutant NEK1 MNs exhibit defects in MT homeostasis and N/C transport that can be restored by PTX

**Fig. 6.**
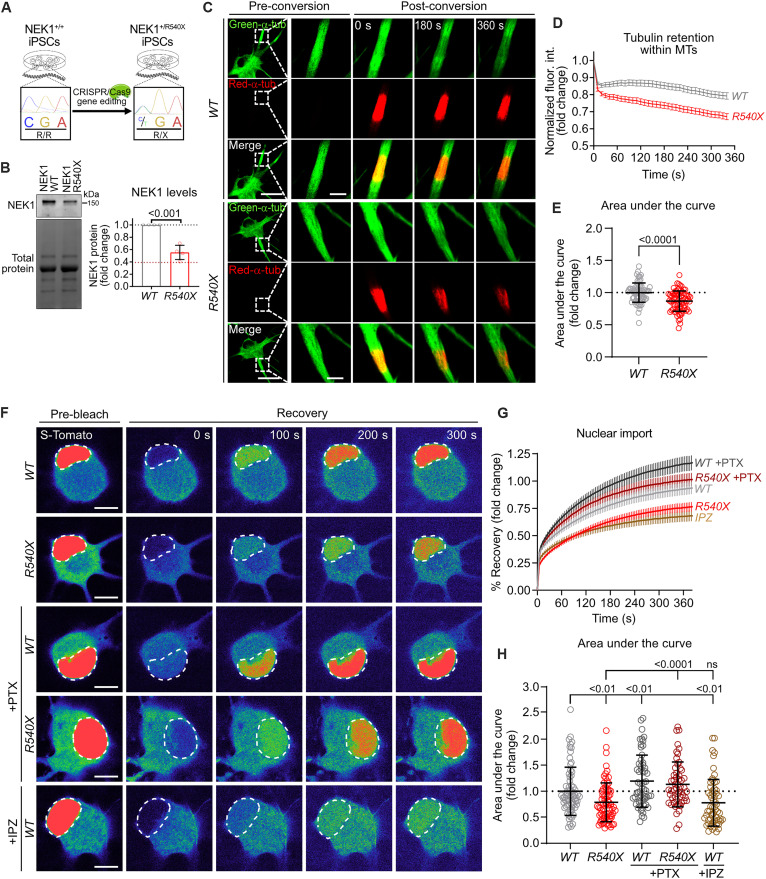
MNs harboring the *NEK1-*ALS mutation R540X exhibit defects in MT homeostasis and N/C transport that can be rescued by PTX. (**A**) Experimental schematic used to introduce p.R540X into a healthy control iPSC line by CRISPR/Cas9 gene editing. (**B**) Left: Representative WB for NEK1 in MNs derived from parental NEK1-WT and NEK1-R540X iPSC lines. Total protein was used as a loading control. Right: Fold change in NEK1 protein levels in WT and R540X MNs. Circles = biological replicates; gray dotted line = mean NEK1 levels in WT MNs; red dotted line = mean NEK1 fold change in *siNEK1*-treated MNs. *n* = 2 independent differentiations. (**C**) Representative confocal images showing tubulin mobility assay in WT and R540X MNs. Dashed lines = inset region magnified in the right time-course images. Scale bars, 25 μm and 5 μm (inset). (**D**) Persistence of photoconverted mEos3.2-TUBA1B fluorescence over time. *n* = 3 independent differentiations; data are represented as mean ± SEM. (**E**) Fold change in the AUC from the line plot shown in (D) in WT and R540X MNs. Circles = individual cells; dotted line = mean AUC in WT MNs. *n* = 3 independent differentiations. (**F**) Representative confocal images showing nuclear import FRAP assay in WT and R540X MNs following vehicle or PTX treatment. IPZ-treated WT MNs are included as a control. Dashed lines = photobleached region (nucleus). Scale bars, 10 μm. (**G**) Percent recovery of reporter fluorescence intensity in the photobleached region of the nucleus over time. *n* = 3 independent differentiations; data are represented as mean ± SEM. (**H**) Fold change in the AUC from line plot shown in (G). Circles = individual cells; dotted line = mean AUC in WT MNs. *n* = 3 independent differentiations. All individual *P* values are shown above comparisons. Data are represented as mean ± SD unless otherwise noted.

Next, we sought to investigate how NEK1 haploinsufficiency affects MN pathobiology in the context of a *NEK1* mutation observed in ALS patients. To this end, we obtained a pair of iPSC lines, one parental line and one line with a heterozygous *NEK1* R540X mutation introduced by CRISPR/Cas9 gene editing (line NEK1-WT, NEK1-R540X; table S1; see Materials and Methods) (*64*), which causes a premature termination codon and renders the mutant transcript susceptible to nonsense-mediated decay (Fig. 6A). The R540X genetic variant has been reported to be present in ALS patients in multiple studies and is extremely rare in the general population [minor allele frequency (MAF) = 0.00001] (*6*, *8*). We validated that the edited, mutant *NEK1* iPSC line and the parental isogenic control retained a normal karyotype and canonical markers of pluripotency (fig. S6, A and B). We also examined differentiated day 50 MNs (fig. S6, C and D) and found that the mutation causes a significant 40 to 60% reduction in full-length NEK1 protein levels, similar to the reduction achieved by siRNA treatment (*P* < 0.0001) (Fig. 6B).

To assess the effect of the ALS-associated patient *NEK1* mutation on the MT cytoskeleton, we performed live-cell imaging of tubulin mobility in mEos3.2-TUBA1B–expressing R540X and isogenic control MNs, using the stimulation parameters and controls described above (Fig. 6, C to E, and fig. S6, E to G). Similar to the effects observed in *siNEK1*-treated control MNs, the NEK1-R540X MNs showed a significant reduction in TUBA1B retention within MTs compared to isogenic controls (*P* < 0.0001) (Fig. 6, C to E). Treatment with PTX significantly increased TUBA1B retention in both genotypes and rescued deficits observed in untreated *R540X* MNs when compared to isogenic controls (WT versus R540X*: P* < 0.001, WT versus WT + PTX: *P* < 0.01, R540X versus R540X + PTX: *P* < 0.0001) (fig. S6F).

To evaluate the effects of the R540X mutation on N/C transport, we measured active nuclear import in differentiated mutant and isogenic control MNs using the cNLS-tdTomato-NES reporter as described above. NEK1 mutant MNs demonstrated a significant reduction in nuclear import rate (*P* < 0.01), with an effect size similar to that of the IPZ control treatment (Fig. 6, F to H). We also found that PTX treatment of mutant NEK1 MNs ameliorated the NEK1-related deficit in nuclear import (*P* < 0.0001) (Fig. 6, F to H), analogous to the effect observed in the siRNA model (Fig. 5, D to F). These effects were observed in the absence of any significant differences in tdTomato reporter expression across genotypes and treatment conditions (fig. S6H). In these experiments, the effect of PTX was not specific to mutant MNs, as even control MNs showed a significant increase in nuclear import levels (*P* < 0.01) (Fig. 6, F to H). Critically, however, PTX also caused a significant increase in NEK1 protein levels exclusively in control MNs (fig. S6, I and J), suggesting that the enhanced nuclear import might be downstream of NEK1 up-regulation. To directly test this hypothesis, we treated control MNs with PTX and blocked NEK1 kinase activity by using the small-molecule inhibitor. We found that the N/C distribution of the tdTomato reporter was reduced back to vehicle control levels by enzymatic inhibition of NEK1 (PTX versus PTX + NEK1i: *P* = 0.002, Vehicle versus PTX + NEK1i, *P* > 0.9999) (fig. S6, K and L). These results highlight the importance of NEK1 activity for modulating nuclear import, whether it be through up-regulation of NEK1 protein levels after PTX treatment or reduction by enzymatic inhibition. Together, these data demonstrate that a recurrent ALS-associated NEK1 mutation causes an impairment in MT homeostasis and nuclear import, and that stabilizing MTs facilitates increased nuclear import levels in mutant MNs.

### *Niki*, the *Drosophila* homolog of *NEK1*, is essential for motor function and survival in vivo

**Fig. 7.**
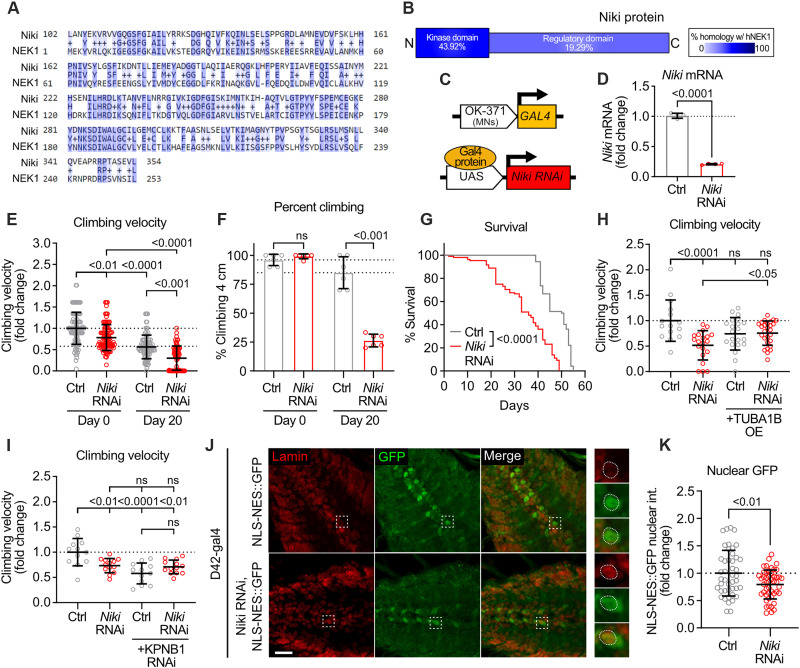
Reduction of the *NEK1* homolog *Niki* causes motor dysfunction and reduced life span in vivo. (**A**) Alignment of *Niki* (*Drosophila*) and *NEK1* (human) amino acids. Dark blue = conservation of amino acid, light blue = conservation of amino acid properties. (**B**) Homology between Niki and human NEK1 in the kinase domain and the regulatory domain. (**C**) Schematic showing genetic paradigm for *Niki* RNAi flies. (**D**) Fold change of *Niki* mRNA in control and *Niki* RNAi flies. *n* = 4 independent experiments. (**E**) Fold change in climbing velocity (cm/s) of control and *Niki* RNAi flies on days 1 and 20. *n* = 69 to 105 flies. (**F**) Fold change in the percent flies climbing 4 cm in 20 s in control and *Niki* RNAi flies on days 1 and 20. *n* = 6 experiments. (**G**) Kaplan-Meier survival curve of control flies and *Niki* RNAi flies. *n* = 70 to 80 flies. (**H**) Fold change in climbing velocity (cm/s) in control and *Niki* RNAi flies (left) compared to control and *Niki* RNAi flies crossed with TUBA1B-overexpressing flies (right) on day 20. *n* = 15 to 27 flies. (**I**) Fold change in climbing velocity (cm/s) in control and *Niki* RNAi flies (left) compared to control and *Niki* RNAi flies crossed with *KPNB1* RNAi flies (right) on day 20. *n* = 12 to 13 flies. (**J**) Representative images of *Drosophila* larval brain of control and *Niki* RNAi flies crossed with flies expressing an NLS-NES-GFP reporter (D42-gal4 MN driver) immunostained for Lamin (red) and GFP (green). Dashed box = magnified region shown in right inset images. Dashed circles = nucleus. Scale bar, 20 μm. (**K**) Fold change in nuclear intensity of the NLS-NES-GFP reporter in control and *Niki* RNAi flies. *n* = 44 to 48 cells from 3 to 5 larvae. All individual *P* values are shown above comparisons. Data are represented as mean ± SD unless otherwise noted. For all plots, circles = individual flies or biological replicates; dotted line = mean of control flies.

Finally, to assess the relevance of NEK1 loss of function in the context of an intact nervous system, we developed a *Drosophila* in vivo model. We found that the fly gene *Niki* shares substantial sequence homology with human *NEK1*, particularly within the kinase domain of these proteins (Fig. 7, A and B). On the basis of this premise, we generated transgenic flies expressing RNAi targeting *Niki* under the OK371-GAL4 driver, which drives expression exclusively in MNs (Fig. 7C) (*65*). Quantitative reverse transcription polymerase chain reaction (RT-PCR) analysis validated the effective knockdown of *Niki* in vivo (*P* < 0.0001) (Fig. 7D). This knockdown elicited a motor phenotype, as measured by climbing velocity, with both young and adult day 20 flies exhibiting a significant reduction in climbing velocity relative to controls (day 0, *P* < 0.01; day 20, *P* < 0.001) (Fig. 7E), as well as an age-dependent reduction in the percent of flies able to climb 4 cm within 20 s (day 20, *P* < 0.0001) (Fig. 7F). Moreover, *Niki*-RNAi flies exhibited a marked age-dependent survival deficit relative to controls as measured by a Kaplan-Meier survival curve (*P* < 0.0001) (Fig. 7G). These data strongly support the notion that *Niki* is essential for motor function and survival in vivo. To investigate the functional relevance of MT homeostasis in this *Drosophila* model, we obtained an overexpression line for human TUBA1B and performed a genetic interaction experiment. We found that while expression of TUBA1B in a control background did not significantly affect motor function, it caused a moderate but significant rescue in climbing velocity in *Niki* RNAi flies (*P* < 0.05) (Fig. 7H). To explore whether *Niki* functions similarly to NEK1 in the regulation of nuclear import, we crossed control and *Niki* RNAi lines with a fly line engineered to express an RNA interference (RNAi) targeting the *Drosophila* homolog of KPNB1. Reduction of KPNB1 did not cause a significant additive motor phenotype to that observed in *Niki* RNAi flies alone (*P* = 0.3571), suggesting that these two proteins may function in the same pathway (Fig. 7I). Last, to directly investigate the effects of *Niki* loss of function on N/C transport, we used a fly model that overexpresses an NLS-NES-GFP reporter in MNs (*65*) and crossed it with *Niki* RNAi flies under the D42-gal4 driver. Using brain tissue sections, we found that *Niki* RNAi flies exhibited a significant reduction in the nuclear intensity of the GFP reporter compared to controls (*P* < 0.01) (Fig. 7, J and K). This result is in accordance with findings in iPSC-derived MNs and suggests that *Niki* loss of function in vivo impairs cNLS nuclear import.

## DISCUSSION

Heterozygous loss-of-function variants in *NEK1* constitute one of the most common genetic causes of ALS (*2*, *3*). However, how NEK1 haploinsufficiency affects MN homeostasis is not well understood. To address this critical gap in knowledge, we identified NEK1 interacting partners and developed human iPSC-based models to interrogate functional defects in NEK1-deficient spinal MNs and *Drosophila* models. We found that NEK1 is essential for motor control in vivo, that it regulates MT homeostasis and N/C transport, and that stabilizing the MT cytoskeleton in MNs improves these defects.

While nonsense variants are unequivocally linked to ALS, the role of rare missense variants in *NEK1* in disease is unclear. Several recent studies focused on identifying patients carrying mutations in multiple ALS genes have described the occurrence of missense *NEK1* variants along with mutations in *SOD1*, *TDP-43*, and more frequently a repeat expansion in *C9orf72* (*9*, *11*, *12*). In most cases, these missense variants are predicted to elicit loss-of-function effects, but this remains empirically undefined. Our functional characterization of NEK1 deficiency, as modeled by a nonsense mutation, an siRNA knockdown approach, and NEK1 kinase inhibition in different genetic backgrounds, suggests that haploinsufficiency can drive disease mechanisms alone. It is likely that partial loss of function can act as a strong modifier converging on the established defects of mutant C9orf72 on the N/C pathway, or of SOD1 and TDP-43 on the cytoskeleton. A detailed and systematic characterization of how missense *NEK1* variants affect kinase activity, protein-protein interaction profile, and the function of downstream pathways, such as the ones we describe here, will lead to a better understanding of the role that *NEK1* genetic heterogeneity plays in ALS disease pathogenesis.

Our proteomics-based interactome and protein abundance experiments highlight a link between NEK1 and the ALS-associated pathways of cytoskeletal homeostasis and N/C transport. These findings are in strong accordance with two previously reported NEK1 interactome analyses that also found several proteins that function in the pathways we identified, including components of the cytoskeletal network and the NPC (*6*, *66*). There was little overlap in specific identified proteins across the three studies likely due to methodological differences. Notably, we did not detect C21ORF2 in our experiments, which is another ALS-associated protein previously shown to interact with and functionally cooperate with NEK1 (*67*). Neither NEK1 interactors nor proteins with NEK1-dependent expression levels were functionally enriched for the DDR pathway in our experiments. The cytoskeleton is widely reported to be disrupted in genetic and sporadic ALS (*2*–*5*), while defective N/C transport, first described in *C9orf72*-associated ALS, appears to be a common feature of several ALS subtypes and other neurodegenerative diseases (*68*, *69*).

We found that NEK1 phosphorylates TUBA1B and KPNB1 in vitro and that reduction in NEK1 levels or enzymatic inhibition of NEK1 modulates their homeostasis and localization, respectively. Both MTs and N/C transporters are known to be regulated by posttranslational modifications that affect their localization and function (*23*, *70*, *71*). Tubulin has several known phosphorylation sites, although many of the corresponding kinases remain unknown (*23*). MT chemical modifications confer key properties to MTs, including localization, subtype, and which motors preferentially traffic along the filament (*23*, *72*). N/C transporter phosphorylation is less well characterized, but phosphorylation has been shown to alter cargo import (*71*). Determining the precise amino acid sites that are phosphorylated by NEK1 will be critical in the future. It will not only provide insight into the regulatory mechanisms of these pathways in postmitotic neurons but also allow for the assessment of the broader relevance of potential defects in posttranslational control of cytoskeletal and N/C transport dynamics across other genetic ALS subtypes and sporadic disease.

Our findings connecting NEK1 with TUBA1B turnover are consistent with the role of NEK1 protein in nonneuronal cells, where it resides in the centrosome, which acts as an MT organizing center (*18*, *20*, *38*, *73*–*75*). Given the effects on MT stability and polymerization, we focused on the downstream functional ramifications related to axonal outgrowth and regeneration. However, MTs play fundamental roles in a range of other neuronal pathways including axonal transport and cytostructural integrity (*47*, *76*, *77*) and the full spectrum of MT-related dysfunction caused by NEK1 mutations remains to be determined.

The importance of MT homeostasis in ALS pathobiology was further underscored by the recent discovery that MT-binding protein STMN2 is a direct and prominent target for TDP-43 regulation (*48*, *49*). Almost all ALS cases are characterized by the loss of nuclear and concomitant cytosolic aggregation of TDP-43. One functional ramification of this event is mis-splicing and down-regulation of STMN2, which plays a fundamental role in motor axon development and regeneration and can ameliorate TDP-43–related deficits in neurite outgrowth (*48*, *49*). We found that down-regulation or enzymatic inhibition of NEK1 compromised axonal regrowth in a manner that is very similar to down-regulation of STMN2 or TDP-43 (*48*, *49*). We also observed that NEK1 localizes in the tips of axonal outgrowths similarly to what has been shown for STMN2 (*48*, *49*). The convergence of both proteins on the MT cytoskeleton renders the question of whether there is overlap on their specific mechanisms of regulating MT homeostasis.

Several lines of evidence support the hypothesis that cytoskeletal defects are central to ALS pathophysiology. First, mutations in several genes that are directly involved in cytoskeletal homeostasis including *ALS2*, *DCTN1, KIF5A, NF-L, NF-H, PFN1, PRPH, SPAST*, and *TUBA4A* can cause or are associated with an increased incidence of ALS (*78*–*90*). Our work calls for the addition of *NEK1* to this list. Second, ALS patient pathology is characterized (although not uniquely) by several signs of disrupted cytoskeletal dynamics including axonal aggregates of hyperphosphorylated neurofilament, MTs, and transport cargo, such as vesicles, lysosomes, and mitochondria (*91*–*95*). Third, disruptions in cytoskeletal structure and MT-dependent axonal transport are an early and predominant neuropathological event in a wide variety of genetic models of ALS (*96*–*106*). Experimental evidence based on mutant mouse models has shown that MT dysfunction may precede neurodegeneration and motor deficits in vivo (*107*, *108*). One study found that the MT-stabilizing drug noscapine restored MT dynamics and prolonged life by as much as 26% in mutant *SOD1^G93A^* mice (*109*).

Our findings demonstrate that NEK1 can independently modulate MT dynamics and N/C transport, while the effects of the MT-stabilizing drugs PTX and Lau highlight an association between these pathways. These results are in accordance with two recent reports focused on ALS and frontotemporal dementia (FTD)–related mutations in *PFN1* and *MAPT,* respectively, which showed a connection between disrupted elements of the cytoskeleton and different aspects of N/C trafficking integrity (*110*, *111*). The precise mechanistic link between the cytoskeleton and N/C transport remains unclear. Nevertheless, the fact that NEK1 acts as a pleiotropic kinase that can modulate both of these pathways that are considered critical for MN homeostasis and contribute to ALS pathophysiology renders this kinase a formidable therapeutic candidate.

## MATERIALS AND METHODS

### Experimental design

The overall objective of the study is to determine the impact of NEK1 loss of function in human MNs. We describe below the materials, methods, and statistical approaches that we used to execute experiments.

### Cell culture conditions

Following procedures we recently described in Ortega *et al.* (*112*), HEK293FT cells were grown in Dulbecco’s modified Eagle’s medium (DMEM) (Corning) supplemented with GlutaMAX (Gibco) and 10% fetal bovine serum (FBS; VWR). HEK293FT cells were dissociated by incubating for 5 min with trypsin-EDTA (Gibco) at 37°C. iPSCs (table S1) were maintained on Matrigel (BD Biosciences) with mTeSR1 medium (STEMCELL Technologies) and passaged on a weekly basis using 1 mM EDTA or Accutase (Sigma). All cell cultures were maintained at 37°C, 5% CO_2_ without antibiotics and tested regularly for mycoplasma. All cell lines used in the study were mycoplasma-free.

### Stem cell cultures and MN differentiation

MN differentiations were performed as described previously (*36*, *112*, *113*). In brief, 70% confluent iPSC cultures were dissociated with Accutase and plated with mTeSR1 and 10 μM ROCK inhibitor (Y-27632, DNSK International) at a density of 100,000 cells/cm^2^. The following day, mTeSR1 was replaced with N2B27 medium (50% DMEM:F12, 50% Neurobasal, supplemented with nonessential amino acids (NEAAs), GlutaMAX, N2, and B27; Gibco) supplemented with a cocktail of small molecules that induces the generation of spinal neural progenitors: 10 μM SB431542 (DNSK International), 100 nM LDN-193189 (DNSK International), 1 μM retinoic acid (RA; Sigma), and 1 μM Smoothened Agonist (SAG; DNSK International). The culture medium was changed daily for 6 days and then switched to N2B27 medium supplemented with 1 μM RA, 1 μM SAG, 5 μM DAPT (DNSK International), and 4 μM SU5402 (DNSK International) to generate postmitotic spinal MNs. Neural cultures were fed daily with this medium for 7 days and then dissociated using TrypLE Express (Gibco) supplemented with deoxyribonuclease (DNase) I (Worthington). Differentiated MN cultures were plated onto precoated Matrigel-coated surfaces (BD Biosciences) and maintained in Neurobasal medium supplemented with NEAAs, GlutaMAX, N2, B27, ascorbic acid (0.2 μg/ml), and brain-derived neurotrophic factor (BDNF), ciliary neurotrophic factor (CNTF), and glial cell line-derived neurotrophic factor (GDNF) (10 ng/ml, R&D Systems). For experiments requiring imaging analysis, MNs were seeded on a preplated monolayer of primary mouse glia cells (harvested from P0 mixed male and female pups of the CD1 strain) as described previously (*36*).

### Overexpression, knockdown, and lentiviral transduction

Overexpression, knockdown, and lentiviral transductions were performed following procedures we recently described in Ortega *et al.* (*112*). For overexpression experiments, 60 to 70% confluent HEK293FT cell cultures were transfected with Lipofectamine 2000 transfection reagent (Thermo Fisher Scientific) according to manufacturer guidelines. Briefly, exogenous DNA was mixed with Lipofectamine 2000 (1 μg DNA:4.5 μl Lipofectamine 2000 ratio) in Opti-MEM medium (Gibco) and incubated for 10 min at room temperature before being added to cells. Analyses made on transfected cells were performed 24 to 48 hours after transfection. For transient knockdown experiments, predesigned siRNAs (Ambion Silencer Select) were transfected using Lipofectamine RNAiMAX (Invitrogen) according to manufacturer guidelines. Briefly, for 2 wells of a 24-well plate with 0.5 × 10^5^ to 2 × 10^5^ cells per well, 10 pmol of siRNA was mixed with 3 μl of Lipofectamine RNAiMAX reagent in 100 μl of Opti-MEM medium and incubated for 15 min at room temperature. Cell medium (400 μl) was then added, and 250 μl was distributed per well. Analyses of siRNA transfection experiments were performed 3 to 4 days after transfection. For lentiviral transductions, iPSC-derived MNs were infected with a previously titered viral burden for 48 hours. Transduced cells were analyzed 7 to 15 days after infection.

### Plasmids and lentiviral production

For AP-MS experiments, a pLV-HA-NEK1 lentiviral transfer vector designed to express HA-tagged NEK1 via an EF1α promoter was constructed and purchased from Vector Builder (Vector ID:VB200124-1112xxw). pLV-mEOS3.2-TUBA1B was manufactured by Vector Builder (Vector ID: VB200728-1215vkd), pLenti-EB1-EGFP was a gift from K.-I. Takemaru (Addgene plasmid #118084), and pLenti-cNLS-tdTomato-NES was provided by J. Rothstein (*54*). Lentiviral packaging was performed as described previously (*113*). Briefly, following overnight seeding in 10 cm plates, HEK293FT cells were transfected with lentiviral transfer vectors listed above (12 μg) along with packaging vectors psPax2 (9 μg) and MDG2 (3 μg) using HilyMax transfection reagent according to the manufacturer’s instructions. Cells were incubated for 4 hours at 37°C before a fresh medium change and 48-hour incubation. The medium was then collected daily for an additional 5 days before filtration (40 μm, Nylon, Fisherbrand) and centrifugation (25,000*g*) for 2 hours at 4°C. Resulting viral pellets were then resuspended in DMEM/F12 before use and/or storage at −80°C. Fresh virus was titrated in neurons using a dilution curve, and expression levels were assessed 7 days after infection.

### Immunocytochemistry

Cells were fixed with 4% paraformaldehyde (PFA) and blocked for 1 hour in phosphate-buffered saline (PBS) containing 10% normal donkey serum (Jackson ImmunoResearch) and 0.1% Triton X-100. Samples were then incubated overnight at 4°C with primary antibodies: ChAT (goat, 1:250, Millipore, catalog no. AB144P, RRID: AB_2079751), MAP2 (chicken, 1:5000, Abcam, catalog no. ab5392, RRID: AB_2138153), human nuclear antigen (mouse, 1:200, Abcam, catalog no. ab191181, RRID: AB_2885016), NEK1 (rabbit, 1:200, Bethyl, catalog no. A304-570A, RRID: AB_2620765), α-tubulin (mouse, 1:500, Millipore, catalog no. 05-829, RRID: AB_310035), phalloidin (two drops per ml, Invitrogen, catalog no. A12379), TUJ1 (rabbit, 1:1000, Sigma-Aldrich, catalog no. T2200, RRID: AB_262133), KPNB1 (mouse, 1:500, Abcam, catalog no. ab2811, RRID: AB_2133989), SAMHD1 (rabbit, 1:250, Proteintech, 12586-1-AP, RRID: AB_2183496), Oct4 (mouse, 1:500, R&D Systems, catalog no. MAB17591, RRID: AB_10719296), and SOX2 (mouse, 1:500, R&D Systems, catalog no. MAB2018, RRID: AB_358009). Primary antibody was removed, and PBS/0.1% Triton was applied for several washes. Samples were then incubated with the appropriate secondary antibodies conjugated to Alexa Fluor 488, Alexa Fluor 555, or Alexa Fluor 647 fluorophores (1:500 or 1:1000, Thermo Fisher Scientific, catalog no. A-21206, RRID: AB_2535792; catalog no. A-21202, RRID: AB_141607; catalog no. A-11056, RRID: AB_2534103; catalog no. A-31572, RRID: AB_162543; catalog no. A-31570, RRID: AB_2536180) for 1 hour at room temperature. Following additional washes, coverslips were mounted, and cell nuclei were labeled by DNA staining with Hoechst mounting medium (Life Technologies). Immunolabeled samples were swapped out for blind image acquisition and analysis.

### Confocal microscopy and quantitative image analysis

Image acquisition for fixed cells was performed on a Nikon W1 dual camera spinning disk confocal microscope with a Plan Apo λ 60× oil immersion objective. Image acquisition was performed through the *z* dimension at 0.3 to 0.5 μm intervals, and individual planes were projected into maximum intensity images. Images used for quantitation and comparison across genotypes or treatments were acquired with an identical exposure time, laser setting, and processing parameters. For each differentiation, fold change values were generated by normalizing the value of each cell to the average value for the siScr or siScr/untreated condition. The mean fluorescence intensity for a region of interest (ROI) consisted of mean pixel intensity per μm^2^, as determined by NIS-Elements Advanced Research v5 software (Nikon software, Northwestern University Center for Advanced Microscopy). For calculation of iPSC differentiation efficiency to MNs, human cells were identified using the human nuclear antigen antibody. Individual frames were then manually counted for percentage of human cells positive for the MN marker ChAT and neuronal marker MAP2. For determination of nuclear intensity, the mean fluorescence intensity was calculated in an ROI drawn using Hoechst signal to define the nucleus within individual cells. For calculation of N/C ratio, the mean fluorescence intensity value was determined for the nucleus (defined using Hoechst signal) and the cytoplasm (defined using MAP2 or ChAT signal) within individual cells. These values were then exported, and the N/C ratio was calculated in Microsoft Excel. For analysis of F-actin levels, mean fluorescence intensity values were determined for the nucleus (defined using Hoechst signal) or cytoplasm (defined using Tuj1 signal) of individual cells, respectively. For analysis of neurite regrowth length, the ends of individual neurites were identified, and lengths were automatically determined in NIS-Elements by their distance to the origin of the axonal chamber. Values were then exported to Microsoft Excel. The following are the minimum number of cells analyzed for each condition within the experiments: For differentiation efficiency, at least 276 cells were analyzed per condition. For F-actin analysis, at least 37 cells were analyzed per condition. For neurite regrowth analysis, at least 553 neurites were analyzed per condition. For KPNB1 nuclear intensity analysis, at least 97 cells were analyzed per condition. For NES-tdTomato-NLS N/C ratio analysis, at least 82 cells were analyzed per condition. For SAMHD1 N/C ratio analysis, at least 95 cells were analyzed per condition.

### Live cell image acquisition and analysis

MNs were maintained at 37°C and 5% CO_2_ using a stagetop incubator (Tokai HIT). Before imaging, the top of the cell culture dish was swapped out and relabeled to allow for image acquisition and analysis in a blind manner for the operator. Imaging was performed on a Nikon A1R+ laser scanning confocal microscope Plan Apo λ 60× oil immersion objective (Northwestern University Center for Advanced Microscopy). Images used for quantitation and comparison across genotypes or treatments were acquired with an identical exposure time, laser setting, and processing parameters. The mean signal intensity for an ROI was the mean pixel intensity per μm^2^ as calculated by NIS-Elements Advanced Research v5 software (Nikon software, Northwestern University Center for Advanced Microscopy).

#### cNLS-tdTomato-NES FRAP analysis

iPSC-derived MNs were transduced with a Lenti-CMV-cNLS-tdTomato-NES construct 7 days before siRNA treatment (Figs. 4 and 5) or 7 days before imaging (Fig. 6). Before photobleaching, three baseline images of neurons expressing the tdTomato reporter were acquired. Individual MN nuclei were photobleached for 30 s (20 iterations of 50 to 70% laser power, 405 nm), and recovery was monitored by imaging at 594 nm every 4 s for up to 6 min after photobleaching. Fluorescence intensity values were normalized to mean baseline signal per nucleus in individual cells, and FRAP curves were generated as mean percent recovery of baseline values over time. For each differentiation, fold change values over time were potted by normalizing % Recovery for each cell to the average % Recovery in the last frame of imaging for the siScr/untreated condition (Figs. 4 and 5) or the NEK1-WT/untreated condition (Fig. 7). Baseline tdTomato levels were determined by measuring mean tdTomato fluorescence intensity within ROIs drawn to encompass the entire MN in the last frame before photobleaching. Fold change values were generated by normalizing the value of each cell to the average value for the siScr or siScr/untreated condition within each differentiation.

#### mEOS3.2-TUBA1B tubulin motility analysis

iPSC-derived MNs were transduced with a photoconvertible mEOS3.2-TUBA1B lentiviral construct 7 days before siRNA treatment (Fig. 3) or 7 days before imaging (Fig. 6). The largest single neurite for each individual neuron was selected for photoconversion and analysis. Before photoconversion, three baseline images of mEOS3.2-expressing neurons were acquired. Photoconversion consisted of three iterations of 10% laser power (405 nm) within a defined ROI with a 5 μm diameter 10 to 20 μm away from the neurite branch point at the soma. Neurons were then imaged every 10 s for up to 6 min, and both green (unconverted) and red (converted) TUBA1B fluorescence was measured. To assess tubulin retention within MTs, red (converted) TUBA1B signal was normalized to green (unconverted) TUBA1B signal at every time point and then normalized to the initial photoconverted signal per neurite.

### Microfluidic isolation of MN neurites and neurite regeneration assay

Neurite regeneration was assessed as previously described (*49*), with minor alterations. To allow for isolation of MN neurites, day 14 MNs were first plated in XonaChip 450 μm microfluidic devices precoated with Matrigel according to the manufacturer’s instructions, at a density of 250,000 neurons per chip. For siRNA experiments, MNs were treated with *siScr* or *siNEK1* (as described above) for 7 days, beginning at day 25 of culture, before axotomy. For NEK1 inhibitor experiments, MNs were cultured to day 32 before a 4-hour treatment with 10 μM NEK1 inhibitor before axotomy. For aspiration-mediated axotomy, prewarmed PBS was pipetted into top well of the axonal side of the XonaChip while simultaneously aspirating from the bottom well (or vice versa) until complete axotomy was confirmed by brightfield microscopy. Complete neuronal growth medium was then replaced in all walls (including vehicle or 10 μM NEK1 inhibitor for inhibitor experiments), and neurites were allowed to regrow for 24 hours. MNs were then fixed using 4% PFA, immunostained, and analyzed as described above.

### Immunohistochemistry and image analysis

Paraffin-embedded tissue sections collected from the motor cortex of two control and two *NEK1*-ALS patients (table S3) were obtained from the VA Biorepository Brain Bank (VABBB) and analyzed by immunohistochemistry for nuclear KPNB1 levels in layer V MNs. Immunohistochemistry was performed as previously described (*114*), with minor alterations. First, slides were deparaffinized and rehydrated through room temperature incubation in the following solutions: 3 × 10 min in xylene, 3 × 5 min in 100% EtOH, 3 × 3 min in 95% EtOH, 1 × 2 min in 75% EtOH, 1 × 2 min in 50% EtOH, and 5 × 1 min in deionized H_2_O. Antigen retrieval was then performed in 1× Antigen Decloaker solution (Biocare Medical) within an electronic pressure cooker for 10 min at 125°C and high (10.2 to 11.6 psi) pressure. Following cooling of sections for 30 min at room temperature, samples were washed five times in deionized water before 1 hour of blocking performed with 1% bovine serum albumin (BSA) in PBS/0.1% Triton X-100 at room temperature in a humidified chamber. Sections were then washed five times with deionized water and incubated with primary antibodies diluted in PBS/1% BSA overnight at 4°C in a humidified chamber. Primary antibodies and dilutions used were as follows: mouse monoclonal anti-KPNB1 (1:100, Abcam, catalog no. ab2811, RRID: AB_2133989) and chicken polyclonal anti-MAP2 (1:1000, Abcam, catalog no. ab5392, RRID: AB_2138153). The following day, sections were washed five times in deionized water and incubated with secondary antibodies diluted in PBS/1% BSA, including donkey polyclonal anti-mouse–Alexa Fluor 488 (1:250, Thermo Fisher Scientific, catalog no.A-21202, RRID: AB_141607) and donkey polyclonal anti-chicken–Alexa Fluor 647 (1:250, Jackson ImmunoResearch, catalog no.703-606-155, RRID: AB_2340380), for 1 hour at room temperature in a humidified chamber. Following five washes with deionized water, sections were incubated for 45 s with 0.3% Sudan black diluted in 70% ethanol and then washed 15 additional times in deionized water. After air drying, slides were mounted using ProLong Diamond Antifade Mountant with DAPI (4′,6-diamidino-2-phenylindole) (Invitrogen) and cured overnight at room temperature. Slides were then imaged using a 60× oil-immersion objective (Plan Apo VC, Nikon) fitted on a Nikon Ti2 inverted confocal microscope with a Yokogawa CSU-W1 spinning disk and Hamamatsu Flash 4 cameras. Layer V MNs were identified using MAP2 as a marker, and z-stacks were acquired using 0.4 μm intervals.

### Coimmunoprecipitation and Western blot

Crosslinking and coimmunoprecipitation were performed as described previously (*115*) with minor adjustments. Before cell lysis, HEK293FT cells expressing HA-tagged NEK1 or iPSC-derived MNs were washed with 1× PBS before crosslinking with the reversible, cell-permeable crosslinker DSP (1 mM, 30 min at room temperature) and quenching with 0.2 M glycine (15 min at room temperature). Cells were then washed with 1× PBS before cell lysis with ice-cold immunoprecipitation buffer [10 mM Hepes (pH 7.6), 100 mM NaCl, 1 mM dithiothreitol (DTT), 10% glycerol, 1% sodium deoxycholate, 0.1% SDS, 1% Triton X-100, protease inhibitor cocktail II, phosphatase cocktail III]. Lysates were then incubated for 10 min on ice, before brief sonication (3 s, 20% amplitude) and clearing by centrifugation (13,000*g* for 10 min at 4°C). For immunoprecipitation of HA-tagged NEK1 or endogenous KPNB1, 5 μg of the following antibodies was coupled to Protein A Dynabeads (Invitrogen, catalog no. 10002D) for 30 min with rotation in PBS with 0.02% Triton X-100: rabbit anti-HA (Abcam, catalog no. ab130275, RRID: AB_11156884), mouse anti-KPNB1 (Abcam, catalog no. ab2811, RRID: AB_2133989), normal rabbit immunoglobulin G (IgG) control (Cell Signaling Technology), and normal mouse IgG control (Santa Cruz Biotechnology). For immunoprecipitation of RFP-tagged TUBA1B, RFP-Trap or SPOT-Trap control nanobody magnetic beads (Chromatek, catalog nos. rtd-10 and etd-10) were used according to the manufacturer’s instructions. Following 1× wash with 0.02% PBS-T (Dynabeads) or tris-buffered saline (TBS) with 0.02% Tween 20 (RFP/SPOT-Trap), 200 μl of corresponding lysates was incubated with magnetic beads for 2 hours at room temperature with constant rotation. Beads were then washed three times with 0.02% PBS-T or 0.02% TBS-T, transferred to a fresh tube, and washed twice with 1× PBS or TBS. Beads were then resuspended in 40 μl of 2× immunoprecipitation sample buffer [120 mM tris (pH 6.8), 0.5% SDS, 20% glycerol, 1% DTT] and heated at 95°C for 10 min to elute bound proteins from beads. Eluants were then directly loaded onto 4 to 20% Mini-PROTEAN TGX Stain-Free Precast Gels (Bio-Rad) for SDS-PAGE/WB analysis or LC-MS analysis (described below).

### Biochemical MT fractionation

Biochemical separation of soluble tubulin and polymerized MT fractions was performed as previously described (*116*) with minor alterations. In brief, day 40 MN lysates were first washed with PBS prewarmed to 37°C and then collected in MT stabilization buffer (100 mM Pipes, 5 mM MgCl_2_, 1 mM EGTA, 30% glycerol, 0.1% NP-40, 0.1% Triton X-100, 0.1% Tween 20, 0.1% β-mercaptoethanol, 100 μM guanosine triphosphate (GTP), 1 mM ATP, 1× phosphatase inhibitor cocktail, 1× protease inhibitor cocktail), also prewarmed to 37°C. Lysates were then homogenized by gentle pipetting and centrifuged for 1 min at 0.1*g* to clear unlysed cells and debris. Cleared lysates were then spun at 100,000*g* for 1 hour at 37°C, and supernatant was collected as the soluble (S) fraction. Pellets (MT fraction) were resuspended in ice-cold 2 mM CaCl_2_ with protease and phosphatase inhibitors and sonicated 3 × 3 s at 30% amplitude. Sonicated pellets were then incubated for 15 min at room temperature to allow MTs to depolymerize and resuspended in Laemmli sample buffer for analysis by WB.

### Affinity purification liquid chromatography mass spectrometry

Cellular extracts were subjected to methanol and chloroform precipitation, and the precipitated protein pellets were solubilized first in 100 μl of 8 M urea for 30 min and then in 100 μl of 0.2% ProteaseMAX (Promega) for an additional 2 hours. The protein extracts were reduced and alkylated, followed by the addition of 300 μl of 50 mM ammonium bicarbonate, 5 μl of 1% ProteaseMAX, and 20 μg of sequence-grade trypsin (Promega). Samples were digested overnight in a 37°C thermomixer (Eppendorf). We used 3 μg of peptides in each Orbitrap Fusion MS analysis.

### Tandem mass spectra analysis

Samples of six independent biological replicates, which included both IgG and HA pull-down of HA-NEK1–transfected cells, were analyzed independently by LC-MS/MS and filtered through Integrated Proteomics Pipeline IP2 version 3, Integrated Proteomics Applications (www.integratedproteomics.com). As we described previously (*112*), each protein identified with the IP2 pipeline was associated with several different measures of abundance used in our analyses, including peptide counts, spectral counts, and normalized spectral abundance factor (NSAF) 78, which takes into account protein length and number of proteins identified in the experiment. When comparing ratios and abundances of a given protein across samples, we used NSAF rank rather than abundance to minimize the effects of differences in sample sizes and stochastic differences between MS analyses. To estimate the number of proteins detected in every experimental condition, we assessed the number of protein entries that displayed detectable NSAF values in either the IgG or HA condition.

### SILAC-based quantitative proteomics analysis

To label iPSC-derived MNs, cells were cultured in medium with or without heavy isotope (^13^C^15^N)–enriched arginine and lysine starting after 16 days of differentiation. The cells were collected after 40 days of differentiation. A sample of the cells was lysed, and proteins were quickly quantified by DC Protein Assay (Bio-Rad). The heavy and light were mixed at a 1:1 ratio based on protein quantification–labeled populations. An aliquot of heavy-labeled population was saved to analyze the labeling efficiency. Samples were lysed in radioimmunoprecipitation assay (RIPA) buffer, incubated for 10 min on ice, sonicated three times (3 s, 60% amplitude), and cleared by centrifugation (15,000*g* for 10 min at 4°C). Proteins were trichloroacetic acid (TCA) precipitated and run on SDS-PAGE gel, and a gel band containing the entire proteome was subjected to in-gel digestion. Gel band was washed in 100 mM ammonium bicarbonate/acetonitrile (AmBic/ACN) and reduced with 10 mM DTT at 50°C for 30 min. Cysteines were alkylated with 100 mM iodoacetamide for 30 min at room temperature protected from light. Gel band was washed again in AmBic/ACN and incubated overnight at 37°C with 600 ng of sequencing-grade trypsin (Promega). Supernatants containing peptides were collected, and the gel was passed through three additional washing steps using 50% ACN/5% FA, 80% ACN/5% FA, and 100% ACN. The supernatant was collected after each wash. The resulting proteins were dried in a vacuum concentrator, reconstituted with 5% ACN/0.1% FA in water, and separated using a linear gradient of solvent A (0.1% formic acid in water) and solvent B (0.1% formic acid in ACN) over 120 min using Dionex UltiMate 3000 Rapid Separation nanoLC (Thermo Fisher Scientific) coupled to the Q Exactive HF Hybrid Quadrupole-Orbitrap Mass Spectrometer (Thermo Fisher Scientific Inc., San Jose, CA). MS raw files were searched using the Mascot search engine (v.2.5.1; Matrix Science, London, UK) against the Swiss-Prot Human database (2019). The proteins identified were filtered for >1 unique peptides using MaxQuant software, which yielded 238 to 4489 proteins depending on the analyzed group. Log_2_ ratio heavy/light (H/L) normalized values were used to carry on the analysis on protein abundance. GO analysis was performed using the online tools DAVID and STRING. All detected proteins were used as background in DAVID analyses, while the entire proteome was used as background in STRING analyses. Sign(log_2_(FC))* log10(formal *P* value) was used to rank the proteins for GSEA, performed using WebGestalt (WEB-based GEne SeT AnaLysis Toolkit). Labeling efficiency was obtained by calculating the ratio of heavy over light peptides detected in the sample treated with heavy isotopes.

### NEK1 kinase activity assay

NEK1 kinase activity was assessed using the NEK1 kinase enzyme system (Promega) and ADP-Glo Kinase Assay (Promega) to measure ATP:ADP conversion, according to the manufacturer’s instructions with minor alterations. Briefly, substrate mixes were prepared in 1× kinase buffer with 50 μM DTT and 100 ng of recombinant GST-His (Novus Biologicals), KPNB1 (Novus Biologicals), TUBA1B (Novus Biologicals), or buffer alone with 83.3 μM ATP (to be diluted to 50 μM in final reaction) and added to small-volume 384-well reaction plates (Greiner Bio-One). Kinase inhibitor (staurosporine, 1 μM final concentration) or vehicle diluted in 5× kinase buffer was then added, followed by the addition of 10 ng of active NEK1 kinase (or buffer alone) diluted in 1× kinase buffer and incubation for 1 hour at room temperature. Following incubation, ATP/ADP standards were prepared and pipetted into adjacent wells. ADP-Glo reagent was then added to all reaction and standard wells and incubated for 40 min at room temperature. Kinase detection reagent was then added, and plates were incubated for an additional 40 min at room temperature before measurement of luminescence (integration time = 0.5 s).

### Drosophila stocks, maintenance, and phenotyping

#### Drosophila melanogaster lines

*Niki* RNAi (*Niki*, stock #16120, RRID: Flybase_FBst0452162) and *Kpnb1* RNAi (*Kpnb1*, stock #CG2637, RRID: Flybase_FBst0479441) lines were obtained from the Vienna Drosophila Resource Center. Control W^1118^ (RRID: BDSC_3605) and OK371-gal4 (RRID: BDSC_26160) lines were described previously (*65*). *HSAP/TUBA1B* (stock #25744, RRID: BDSC_25774), UAS-NLS-NES-GFP (RRID: BDSC_7032), elav-Gal4 (RRID: BDSC_8760), and D42-Gal4 (RRID: BDSC_8816) lines were obtained from the Bloomington Drosophila Stock Center. All *Drosophila* stocks used in this study were maintained on standard cornmeal medium at 25°C or 18°C in light/dark-controlled incubators.

#### Drosophila RNA collection and quantitative PCR

RNA was extracted in triplicate from Niki RNAi and *W^1118^* control adult fly brains using TRIzol (Ambion, catalog no. 15596026) in 1-bromo-3-chloropropane (BCP; Sigma-Aldrich, catalog no. MKCB0830V), as previously described (*117*). Heads were harvested from adult flies, snap-frozen on dry ice, ground in TRIzol, and centrifuged to pellet. BCP was added, and samples were centrifuged again. The upper aqueous layer was used to precipitate RNA with isopropanol, followed by pelleting RNA through centrifugation. RNA pellets were washed with 70% ethanol and allowed to air-dry before resuspending in ribonuclease (RNase)–free water. RNA samples were quantified on a NanoDrop ND-1000 spectrophotometer, and purity was assessed using 260/280 and 260/230 ratios. cDNA was then generated from RNA samples with the Bio-Rad iScript Select cDNA Synthesis Kit (catalog no.170–8897) in a Thermo Hybaid Omn-E PCR machine. All cDNA samples were run on a 96-well plate (Applied Biosystems, catalog no. 4306737) on an Applied Biosystems 7300 Real-Time PCR system with Bio-Rad iQ Supermix (catalog no.170–8862). cDNA-specific PrimeTime qPCR Assay primers (Integrated DNA Technologies) were used for quantitative PCRs (www.idtdna.com). Drosophila glyceraldehyde-3-phosphate dehydrogenase (GAPDH) (dGapdh) was used as a housekeeping control (*65*). The comparative *C*_T_ method was used to analyze results, as previously described (*118*). GraphPad Prism 6 software was used for statistical analyses. Niki primer sequences were as follows: probe, TCCTTTGGCACATAGCGTTCTGGT; forward, TGGCGTGGTCGTGTCTA; reverse, GCGACCGTGGCTATTACG.

#### Climbing assays

Niki RNAi or control (*W^1118^*) was expressed in MNs (OK371-gal4) and aged for 1 or 20 days, and climbing assays were performed as previously described (*65*, *119*). Briefly, adult flies were anesthetized with CO_2_ after 1 or 20 days, transferred to vials, and allowed to recover for 45 min. Flies were knocked to the bottom of vials by tapping against the bench three times, and a video camera was used to record flies climbing up the walls. The percentage of flies that climbed 4 cm in 20 s as well as the velocity (cm/s) of each individual fly were quantified and analyzed using GraphPad Prism 6 software. Six experimental replicates were performed for each group.

#### Survival assay

Niki RNAi or control (*W^1118^*) was expressed in MNs (OK371-gal4), and adult 2-day-old flies (20 flies per vial, 70 to 80 flies) were collected for each experimental group. Flies were transferred to fresh food twice a week. The number of dead flies was counted every day, and survival functions were calculated and plotted as Kaplan-Meier survival curves. Log-rank with Grehan-Breslow-Wilcoxon tests were performed to determine significance differences in survival data between samples using GraphPad Prism 6 software.

#### NLS-NES-GFP N/C reporter assay

Third-instar larvae brains were dissected, fixed, and immunostained as previously described (*119*). Briefly, the animals were dissected in ice-cold PBS, fixed in 4% PFA, and incubated with primary antibody: mouse anti-Lamin Dm0 (Developmental Studies Hybridoma Bank) overnight at 4°C. The following day, the brains were incubated with secondary antibodies and mounted using DAPI Fluoroshield. Images were captured via a Nikon A1 Eclipse T confocal microscope. The relative nuclear intensity of enhanced GFP (eGFP)–positive cells was quantified between the control and sample and compared using an unpaired *t* test.

### WB analysis

Cells were harvested in RIPA buffer [10 mM tris-HCl (pH 8.0), 140 mM NaCl, 1 mM EDTA, 0.1% SDS], supplemented with 1% Triton X-100 (Triton, Sigma-Aldrich), protease inhibitor cocktail III (Millipore), and phosphatase inhibitor cocktail II (Abcam). Lysates were sonicated and protein extracts were separated by SDS-PAGE followed by electrotransfer to nitrocellulose membranes (Bio-Rad). The membranes were blocked for 1 hour at room temperature with 5% nonfat dry milk (Labscientific) in TBS (50 mM tris, 150 mM NaCl, HCl to pH 7.6) with 0.1% Tween 20 (Bio-Rad) (TBS-T), before overnight incubation at 4°C with primary antibodies diluted in TBS-T with 5% BSA (Calbiochem). After several washes in TBS-T, membranes were incubated with their corresponding secondary horseradish peroxidase–conjugated antibodies (1:5000, LI-COR Biotechnology, catalog no. 926-80011, RRID: AB_2721264; catalog no. 926-80010, RRID: AB_2721263) in TBS-T with 5% nonfat milk. Following additional TBS-T washes, signal corresponding to specific proteins was detected by ChemiDoc XRS+ (Bio-Rad), using the SuperSignal West Pico chemiluminescent system (Thermo Scientific). Densitometry analysis for the bands was performed using Image Lab software (Bio-Rad). Primary antibodies used were as follows: mouse anti-NEK1 (1:500, Santa Cruz Biotechnology, catalog no. sc-398813, RRID: AB_2885034), mouse anti–α-tubulin (1:5000, Millipore, catalog no. 05-829, RRID: AB_310035), rabbit anti-GAPDH (1:1000, Cell Signaling Technology, catalog no. 2118, RRID: AB_561053), mouse anti-HA (1:1000, Abcam, catalog no. ab130275, RRID: AB_11156884), rabbit anti–α-tubulin (1:1000, Cell Signaling Technology, catalog no. 2125, RRID: AB_2619646), mouse anti-KPNB1 (1:1000, Abcam, catalog no. ab2811, RRID: AB_2133989), rabbit anti-KPNA2 (1:1000, Proteintech, catalog no. 10819-1-AP, RRID: AB_2265526), mouse anti-IPO5 (1:500, Santa Cruz Biotechnology, catalog no. sc-55527, RRID: AB_2127684), mouse anti-IPO7 (1:500, Santa Cruz Biotechnology, catalog no. sc-365231, RRID: AB_10850405), mouse anti-VIM (1:500, Santa Cruz Biotechnology, catalog no. sc-6260, RRID: AB_628437), rabbit anti-HSPA8 (1:1000, Santa Cruz Biotechnology, catalog no. sc-7298, RRID: AB_627761), mouse anti-Hsp90α/β (1:1000, Santa Cruz Biotechnology, catalog no. sc-13119, RRID: AB_675659), and mouse anti-Hsp70 (1:1000, Santa Cruz Biotechnology, catalog no. sc-32239, RRID: AB_627759).

### CRISPR/Cas9 genome editing of human iPSC line

The NEK1-02-WT iPSC line was edited by The Jackson Laboratory by a ribonucleoprotein (RNP)–based approach. Briefly, 1 million patient iPSCs were electroporated with a mixture of single-guide RNA (sgRNA) and Cas9 in the RNP format, and single-stranded oligodeoxynucleotides (ssODN). A small portion of the cell culture, presumably with mixed population, was subjected to PCR and Sanger sequencing analysis. Once the mixed culture showed repair with qualified homology-directed repair (HDR) efficiency, the transfected cells were subjected to single-cell cloning. After 2 weeks in culture, individual colonies were picked and expanded. A fraction of cells from each clone was collected for genotyping analysis by PCR and Sanger sequencing. Positive clones were further expanded and submitted again for sequencing to confirm desired genotype. iPSC clones were then cryopreserved and shipped to our laboratory.

### Statistical analysis

We performed all statistical analyses using Prism 9 software (GraphPad Software). We classified an independent biological replicate as either an independent transfection experiment or an independent iPSC differentiation performed on a different day. The numbers (*n*) and significance value (*P* value) of each specific experiment are provided in Results, and the statistical test performed for each specific experiment is defined in the corresponding figure legend. For each statistical analysis, we first tested whether sample datasets demonstrated significantly different variances, using the Brown-Forsythe analysis of variance (ANOVA) test, to ensure that pooling of data in each experimental condition was appropriate across independent differentiations. We next tested whether sample data fit a Gaussian distribution using the D’Agostino-Pearson test. For experimental conditions that included *n* = 2 conditions, we performed either an unpaired *t* test (parametric, no significant difference in variance between distributions) or a Mann-Whitney *U* test (nonparametric). For experimental conditions that included at least *n* ≥ 3, we performed a one-way ANOVA followed by Tukey’s post hoc test (parametric), a Kruskal-Wallis rank test followed by Dunn’s correction for multiple comparisons (nonparametric), or a repeated-measures ANOVA followed by Šídák’s multiple comparisons for *Drosophila* assays run on the same flies at multiple time points. For live-cell NES-tdTomato-NLS FRAP experiments, we performed Kruskal-Wallis rank tests followed by the two-stage linear step-up procedure of Benjamini, Krieger, and Yekutieli (nonparametric) to increase statistical power, given the inherently high variability and low-throughput nature of the assay. For imaging experiments measuring a continuous variable at single-cell resolution, we accounted for variability among experimental replicates by normalizing all measurements to the average and SD of the corresponding controls. Detailed statistical approaches for all individual figures can be found below:

Figure 1: (E) *n* = 10 independent differentiations; unpaired *t* test; individual *P* values noted above comparisons. (G) *n* = 2 differentiations where *siScr*-treated cultures were labeled with heavy amino acids and *n* = 2 differentiations where *siNEK1*-treated cultures were labeled with heavy amino acids; GSEA analysis. Individual *q* values for all enriched terms are as follows: Biological Process, from top to bottom: 0.009, 0.04, 0.02, 0.03, 0.04, 0.04, 0.05, 0.03, 0.05, 0.01; Molecular Function, from top to bottom: 0.03, 0.05, 0.09. 0.2, 0.2, 0.3, 0.1, 0.1, 0.08, 0.02.

Figure 2: (C) *n* = 3 reactions; bars represent mean ± SD, one-way ANOVA with Tukey’s correction for multiple comparisons; adjusted *P* values noted above comparisons.

Figure 3: (F) *n* = 4 independent differentiations; bars represent mean ± SD, Mann-Whitney test; individual *P* values noted above comparisons. (I) *n* = 3 independent differentiations; Mann-Whitney test, individual *P* values noted above comparisons. *n* = 3 to 4 independent differentiations; Mann-Whitney test, individual *P* values noted above comparisons.

Figure 4: (B) *n* = 3 independent differentiations; bars represent mean ± SD; Mann-Whitney test, individual *P* values noted above comparisons. (C) *n* = 3 independent differentiations; bars represent mean ± SD; Kruskal-Wallis test with Dunn’s correction, adjusted *P* values noted above comparisons. (F) *n* = 2 patients per disease condition; bars represent mean ± SD; Kruskal-Wallis test with Dunn’s correction, adjusted *P* values noted above comparisons. (J) *n* = 3 independent differentiations; bars represent mean ± SD; Mann-Whitney test, individual *P* values noted above comparisons. (L) *n* = 3 independent differentiations; bars represent mean ± SD; Mann-Whitney test, individual *P* values noted above comparisons. (N) *n* = 3 independent differentiations; bars represent mean ± SD; Mann-Whitney test, individual *P* values noted above comparisons.

Figure 5: (F) *n* = 4 independent differentiations; bars represent mean ± SD, Kruskal-Wallis test with the two-stage step-up procedure of Benjamini, Krieger, and Yekutieli to correct for multiple comparisons; adjusted *P* values noted above comparisons. (I) *n* = 3 independent differentiations; bars represent mean ± SD, Kruskal-Wallis test with the two-stage step-up procedure of Benjamini, Krieger, and Yekutieli to correct for multiple comparisons; adjusted *P* values noted above comparisons.

Figure 6: (B) *n* = 2 independent differentiations; unpaired *t* test, individual *P* values noted above comparisons. (E) *n* = 3 independent differentiations; bars represent mean ± SD, unpaired *t* test, individual *P* values noted above comparisons. (H) *n* = 3 independent differentiations; bars represent mean ± SD, Kruskal-Wallis test with the two-stage step-up procedure of Benjamini, Krieger, and Yekutieli to correct for multiple comparisons; adjusted *P* values noted above comparisons.

Figure 7: (D) *n* = 4 independent experiments; bars represent mean ± SD, unpaired *t* test, individual *P* values noted above comparisons. (E) *n* = 69 to 105 flies; bars represent mean ± SD, Kruskal-Wallis test with Dunn’s correction, adjusted *P* values noted above comparisons. (F) *n* = 6; bars represent mean ± SD, repeated-measures ANOVA with Šídák’s multiple comparisons test, adjusted *P* values noted above comparisons. (G) *n* = 70 to 80 flies, Kaplan-Meier survival analysis, *P* values noted beside comparison. (H) *n* = 15 to 27 flies; bars represent mean ± SD, one-way ANOVA with Tukey’s correction, adjusted *P* values noted above comparisons. (I) *n* = 12 to 13 flies; bars represent mean ± SD, one-way ANOVA with Tukey’s correction, adjusted *P* values noted above comparisons. (K) *n* = 44 to 48 cells from 3 to 5 larvae; bars represent mean ± SD, unpaired *t* test, individual *P* values noted above comparisons.
