# Supplementary material for: Loss of function of the ALS-associated NEK1 kinase disrupts microtubule homeostasis and nuclear import
Source: Sci Adv. 2023 Aug 16;9(33):eadi5548. doi: 10.1126/sciadv.adi5548 (PMC10431718; doi:10.1126/sciadv.adi5548)
Supplement: Supplementary file 1 — Figs. S1 to S6 Tables S1 to S3 Legend for data S1 [file sciadv.adi5548_sm.pdf]

Science Advances
AAAS

Supplementary Materials for

Loss of function of the ALS-associated NEK1 kinase disrupts microtubule homeostasis and nuclear import

Jacob R. Mann *et al.*

Corresponding author: Evangelos Kiskinis, evangelos.kiskinis@northwestern.edu

Sci. Adv. **9**, eadi5548 (2023)
DOI: 10.1126/sciadv.adi5548

The PDF file includes:

Figs. S1 to S6
Tables S1 to S3
Legend for data S1

Other Supplementary Material for this manuscript includes the following:

Data S1

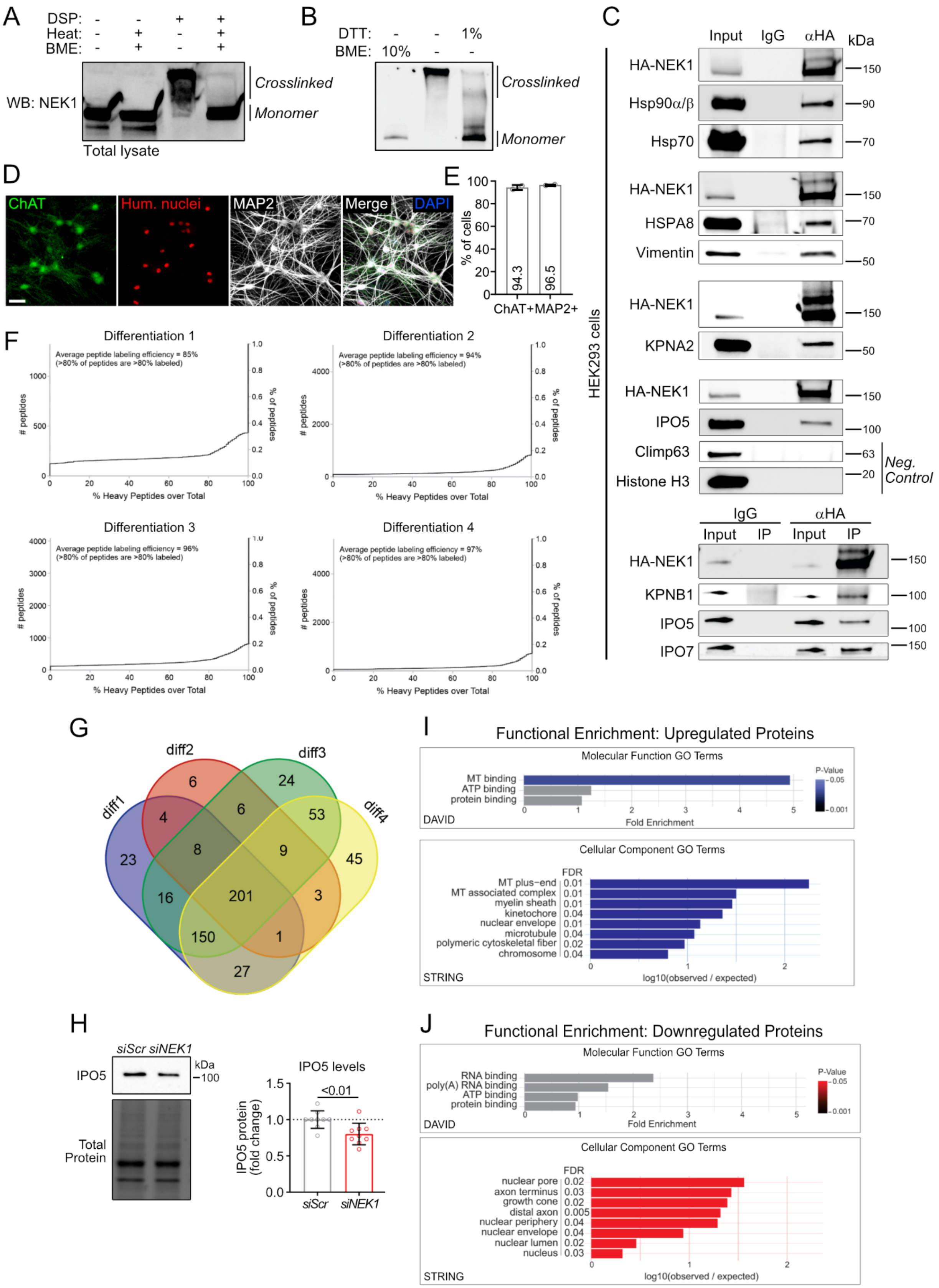
A
DSP: - - + +
Heat: - + - +
BME: - + - +
WB: NEK1
Crosslinked
Monomer
Total lysate
B
DTT: - - 1%
BME: 10% - -
Crosslinked
Monomer
C
Input IgG αHA kDa
HA-NEK1
Hsp90α/β
Hsp70
HSPA8
Vimentin
KPNA2
IPO5
Climp63
Histone H3
Neg. Control
HEK293 cells
IgG αHA
Input IP Input IP
KPNB1
IPO7
150
90
70
50
100
63
20
D
ChAT
Hum. nuclei
MAP2
Merge
DAPI
E
% of cells
94.3
96.5
ChAT+
MAP2+
F
Differentiation 1
Average peptide labeling efficiency = 85%
(>80% of peptides are >80% labeled)
Differentiation 2
Average peptide labeling efficiency = 94%
(>80% of peptides are >80% labeled)
Differentiation 3
Average peptide labeling efficiency = 96%
(>80% of peptides are >80% labeled)
Differentiation 4
Average peptide labeling efficiency = 97%
(>80% of peptides are >80% labeled)
peptides
% of peptides
% Heavy Peptides over Total
G
diff1
diff2
diff3
diff4
H
siScr siNEK1
IPO5
kDa
100
Total Protein
IPO5 levels
<0.01
IPO5 protein (fold change)
siScr
siNEK1
I
Functional Enrichment: Upregulated Proteins
Molecular Function GO Terms
MT binding
ATP binding
protein binding
P-Value
DAVID
Fold Enrichment
Cellular Component GO Terms
FDR
MT plus-end 0.01
MT associated complex 0.01
myelin sheath 0.01
kinetochore 0.04
nuclear envelope 0.01
microtubule 0.04
polymeric cytoskeletal fiber 0.02
chromosome 0.04
STRING
log10(observed / expected)
J
Functional Enrichment: Downregulated Proteins
Molecular Function GO Terms
RNA binding
poly(A) RNA binding
ATP binding
protein binding
P-Value
DAVID
Fold Enrichment
Cellular Component GO Terms
FDR
nuclear pore 0.02
axon terminus 0.03
growth cone 0.02
distal axon 0.005
nuclear periphery 0.04
nuclear envelope 0.04
nuclear lumen 0.02
nucleus 0.03
STRING
log10(observed / expected)

Fig. S1. Proteomics analysis of NEK1 interactors and expression changes converges on microtubule homeostasis and nucleocytoplasmic transport.

(**A**) Representative WB of endogenous NEK1 complexes of high molecular weight in HEK293 cells following DSP crosslinking and native PAGE. (**B**) Representative WB of immunoprecipitated HA-NEK1 following DSP crosslinking and reversal by 1% DTT. (**C**) Representative WBs confirming co-immunoprecipitation of NEK1 interactors (HSP90α/β, HSP70, HSPA8, VIMENTIN, KPNA2, KPNB1, IPO5, and IPO7) identified by LC-MS/MS analysis with HA-tagged NEK1 in HEK293 cells. (**D**) Representative images showing D50 MNs differentiated from control iPSCs and immunostained for ChAT (green), MAP2 (white), human nuclei (red), and DAPI (blue). Scale bar = 50μm. (**E**) Bar plots showing the percentage of human cells positive for the MN marker ChAT (green) and neuronal marker MAP2 (red). (n=2 independent differentiations, bars represent mean ± SD). (**F**) Cumulative line plots demonstrating SILAC labeling efficiency across all four independent differentiation experiments. The graph represents the ratio of heavy/light peptides detected in a labeled sample with the number of peptides (#) and the percent (%) of peptides labeled. (**G**) Venn diagram showing the overlap of proteins identified by LC-MS/MS between n=4 differentiation experiments. (**H**) Left: Representative WB for IPO5 in *siScr*- and *siNEK1*-treated MN cultures. Total protein was measured for loading control. Right: Bar plots showing the fold change in the IPO5/total protein ratio. Circles represent individual samples, and the dotted line marks mean IPO5 levels in *siScr*-treated MNs. (n=4 independent differentiations; unpaired t-test). (**I**) Top: Gene Ontology (GO) analysis using DAVID assessed for significant enrichment in molecular function among the 13 unique proteins upregulated in *siNEK1*-treated MN cultures. Bars represent the fold enrichment for each molecular function term. Bar color represents the p-value according to the given key. The number of upregulated proteins in our dataset found in each category are as follows: MT binding = 4; ATP binding = 3; protein binding = 9. See table S1 for more details. Bottom: STRING interaction network analysis for significant enrichment in cellular component among the 37 proteins upregulated in all four experiments in *siNEK1*-treated MN cultures. Blue bars represent the strength of enrichment (log10(observed/expected)). False discovery rate (FDR) values for each molecular function are displayed on the left. (**J**) Top: Gene Ontology (GO) analysis using DAVID assessed for significant enrichment in molecular function among the 15 unique proteins downregulated in *siNEK1*-treated MN cultures. Bars represent the fold enrichment for each molecular function term. Bar color represents the p-value according to the given key. The number of upregulated proteins in our dataset found in each category are as follows: RNA binding = 4; poly(A) RNA binding = 8; ATP binding = 3; protein binding = 10. See table S1 for more details. Bottom: STRING interaction network analysis for significant enrichment in cellular component among the 29 proteins downregulated in all four experiments in *siNEK1*-treated MN cultures. Red bars represent the strength of enrichment (log10(observed/expected)). False discovery rate (FDR) values for each molecular function are displayed on the left.

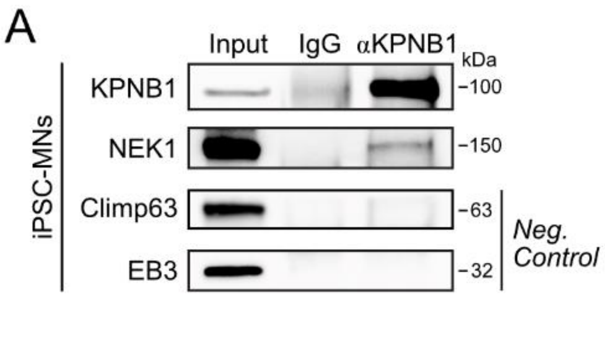

A
Input
IgG
αKPNB1
kDa
iPSC-MNs
KPNB1
NEK1
Climp63
EB3
100
150
63
32
Neg.
Control
B
Input
Ctrl-
TRAP
RFP-
TRAP
kDa
iPSC-MNs
α-tubulin
NEK1
TDP-43
75
50
150
50
Neg.
Control

Fig. S2. KPNB1 and TUBA1B interact with endogenous NEK1 in motor neurons.

(**A**) Top: Representative WB using D40 control iPSC-MN lysates immunoprecipitated with IgG (control) or αKPNB1 and probed for NEK1 and negative control proteins Climp63 and EB3. (**B**) Representative WB using D40 RFP-TUBA1B iPSC-MN lysates immunoprecipitated with αSPOT beads (control) or αRFP beads and probed for NEK1 and negative control protein TDP-43.

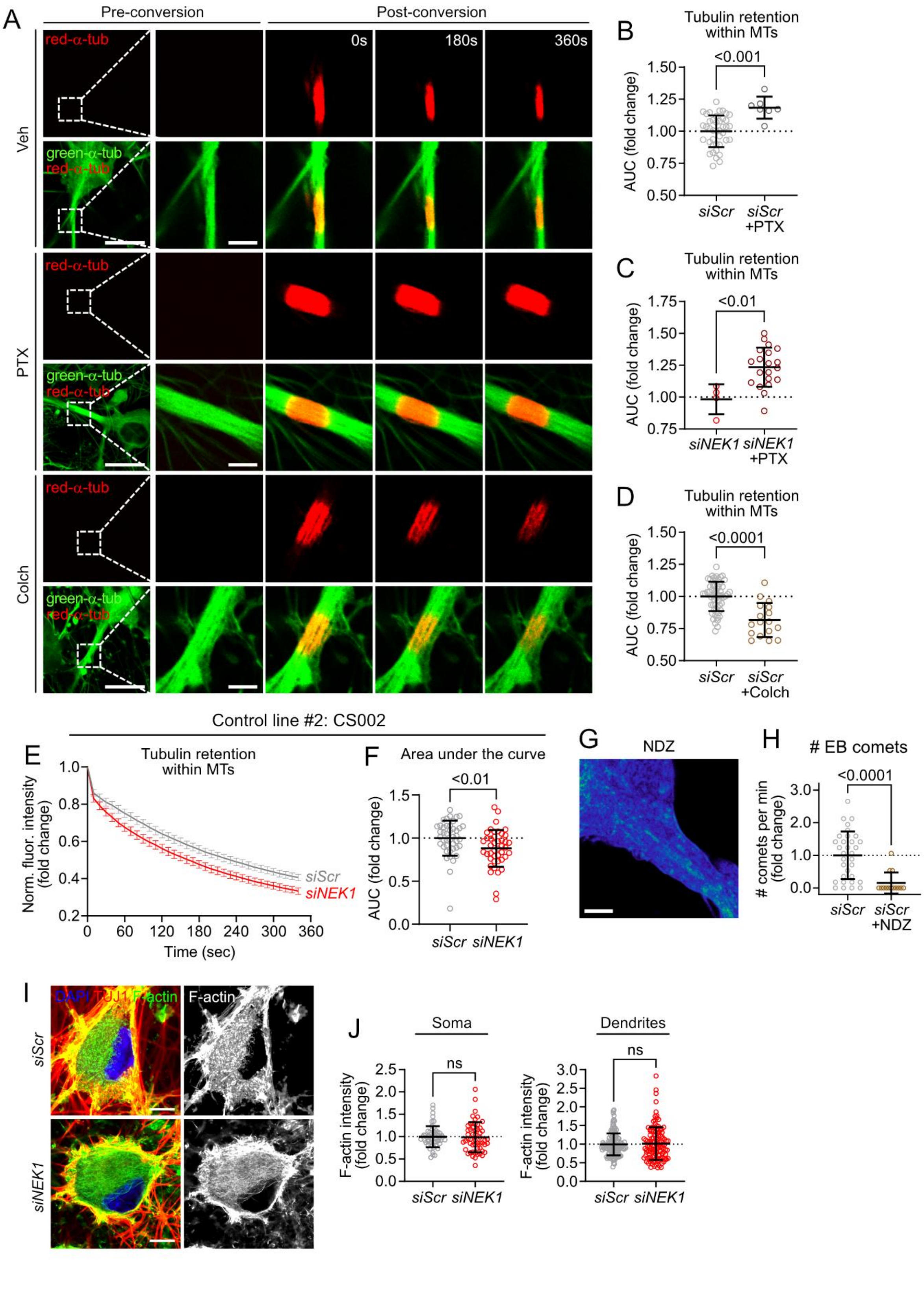

A
Pre-conversion
Post-conversion
0s
180s
360s
red-α-tub
green-α-tub
Veh
PTX
Colch
B
Tubulin retention within MTs
<0.001
AUC (fold change)
siScr
siScr +PTX
C
Tubulin retention within MTs
<0.01
siNEK1
siNEK1 +PTX
D
Tubulin retention within MTs
<0.0001
siScr +Colch
Control line #2: CS002
E
Tubulin retention within MTs
Norm. fluor. intensity (fold change)
Time (sec)
siScr
siNEK1
F
Area under the curve
<0.01
G
NDZ
H
EB comets
<0.0001
comets per min (fold change)
siScr +NDZ
I
DAPI TUJ1 F-actin
F-actin
J
Soma
Dendrites
ns
F-actin intensity (fold change)

Fig. S3. Reduction of NEK1 levels in motor neurons disrupts microtubule homeostasis.

(**A**) Representative live confocal images showing tubulin mobility assay in vehicle-, PTX-, and NDZ-treated MN cultures. Dashed lines mark the inset region magnified in the right time-course images. Scale bars: 25µm, 5µm (inset). (**B**) Dot plot of the fold change in the area under curve (AUC) from tubulin motility assay (as in Fig. 2F) in *siScr*-treated MN cultures with and without PTX (50nM) treatment. Individual cells are displayed as circles and dotted line marks the mean AUC in control MNs. (n=1 differentiation; bars represent mean ± SD, unpaired t-test; individual p-values noted above comparisons). (**C**) Dot plot of the fold change in the area under curve (AUC) from tubulin motility assay (as in Fig. 2F) in *siNEK1*-treated MN cultures with and without PTX (50nM) treatment. Individual cells are displayed as circles and dotted line marks the mean AUC in control MNs. (n=1 differentiation; bars represent mean ± SD, unpaired t-test; individual p-values noted above comparisons). (**D**) Dot plot of the fold change in the area under curve (AUC) from tubulin motility assay (as in Fig. 2F) in *siScr*-treated MN cultures with and without Colch (1µM, 2hr) treatment. Individual cells are displayed as circles and dotted line marks the mean AUC in control MNs. (n=2 independent differentiations; bars represent mean ± SD, unpaired t-test; individual p-values noted above comparisons). (**E**) Line plot showing the persistence of photoconverted mEos3.2-TUBA1B fluorescence (normalized to green fluorescence intensity) in the photoconverted 5 µm region of *siScr*- and *siNEK1*-treated MN neurites differentiated from a second control iPSC line (CS002) over time. The average baseline fluorescence before photoconversion was set as 100%. (n=3 independent differentiations; lines and error bars represent mean ± SEM). (**F**) Dot plot of the fold-change in the area under curve (AUC) from line plots shown in (E) in *siScr*- and *siNEK1*-treated MN cultures. Individual cells are displayed as circles and dotted line marks the mean AUC in control MNs. (n=3 independent differentiations; bars represent mean ± SD, Mann-Whitney test; individual p-values noted above comparisons). (**G**) Representative maximum intensity in time projections of total MT trajectories (cumulative over a 120 second interval) from EB1-GFP live confocal imaging of NDZ-treated MNs. Scale bar: 10µm. (**H**) Dot plot displaying the number of EB comets per minute in *siScr*-treated MN cultures with and without NDZ (10µM, 4hr) treatment. Individual cells are represented as circles. (n=1 differentiation; Mann-Whitney test, individual p-values noted above comparisons). (**I**) Representative confocal images of *siScr*- and *siNEK1*-treated MNs immunolabeled with F-actin (green), TUJ1 (red) and DAPI (blue). Scale bars: 10µm. (**J**) Dot plots showing the fold-change in F-actin levels in the soma (left) and proximal dendrites (right) of *siScr*- and *siNEK1*-treated MN cultures. Individual cells are displayed as circles and dotted line marks mean F-actin in control MNs. (n= 2 independent differentiations; bars represent mean ± SD; Mann-Whitney test, individual p-values noted above comparisons).

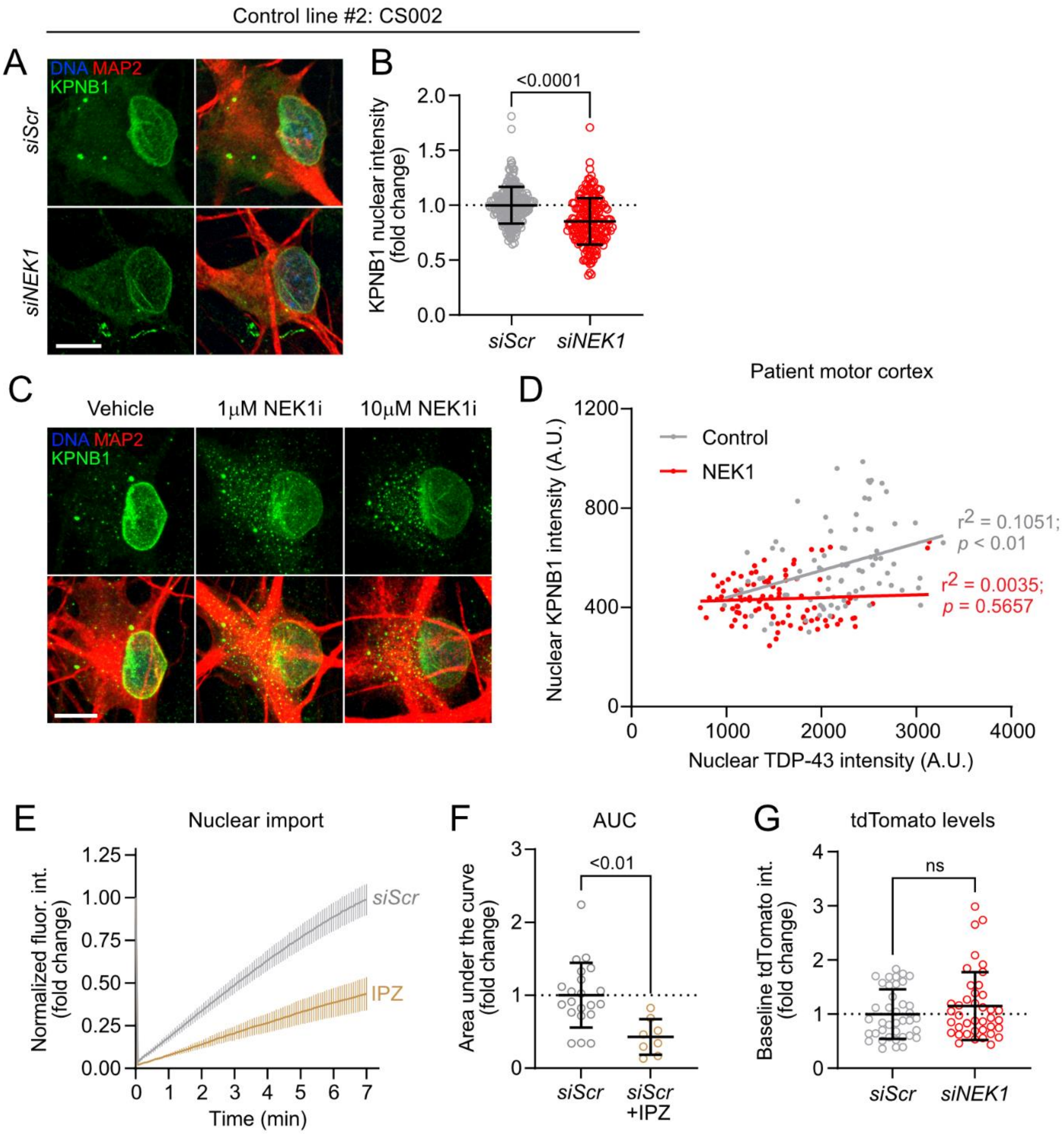

Control line #2: CS002
A
DNA MAP2
KPNB1
siScr
siNEK1
B
<0.0001
KPNB1 nuclear intensity (fold change)
siScr
siNEK1
C
Vehicle
1μM NEK1i
10μM NEK1i
DNA MAP2
KPNB1
D
Patient motor cortex
Control
NEK1
r2 = 0.1051; p < 0.01
r2 = 0.0035; p = 0.5657
Nuclear KPNB1 intensity (A.U.)
Nuclear TDP-43 intensity (A.U.)
E
Nuclear import
siScr
IPZ
Normalized fluor. int. (fold change)
Time (min)
F
AUC
<0.01
Area under the curve (fold change)
siScr
siScr +IPZ
G
tdTomato levels
ns
Baseline tdTomato int. (fold change)
siScr
siNEK1

Fig. S4. Reduction of NEK1 levels in motor neurons perturbs nucleocytoplasmic transport. (**A**) Representative images of *siScr*- and *siNEK1*-treated MNs differentiated from a second control iPSC line and immunolabeled for KPNB1 (green), MAP2 (red) and DAPI (blue). Scale bar = 10μm. (**B**) Dot plot showing the fold change of nuclear KPNB1 fluorescence intensity in *siScr*- and *siNEK1*-treated MNs differentiated from a second control iPSC line (CS002). Individual cells are displayed as circles and dotted line marks the mean in control MNs. (n=3 independent differentiations; bars represent mean ± SD; Mann-Whitney test, individual p-values noted above comparisons). (**C**) Representative images of vehicle- and NEK1 inhibitor-treated iPSC-MNs immunolabeled for KPNB1 (green), MAP2 (red) and DAPI (blue). Scale bar = 10μm. (**D**) Dot plot showing the correlation between KPNB1 and TDP-43 nuclear intensities in the motor cortex neurons shown in Fig. 4D-F. There is a significant positive correlation between KPNB1 and TDP-43 nuclear intensities in control patient neurons (gray), while little correlation exists in NEK1-ALS patient neurons (red). (n = 2 patients per disease condition, 46-61 neurons per condition; individual neurons are displayed as dots). (**E**) Line plot showing the percent (%) recovery (red fluorescence intensity) in the photobleached region of the nucleus of *siScr*-treated MNs with and without IPZ treatment over time. The average baseline fluorescence intensity before photobleaching was set as 100%. (n=2 independent differentiations; lines and error bars represent mean ± SEM). (**F**) Dot plot showing the fold change in the area under curve (AUC) from line plots shown in (D). Individual cells are displayed as circles and dotted line marks the mean AUC in control MNs. (n=2 independent differentiations; bars represent mean ± SD; unpaired t-test, individual p-values noted above comparisons). (**G**) Dot plot showing the fold change in whole-cell NES-tdTomato-NLS fluorescence intensity at baseline prior to photobleaching. Individual cells are displayed as circles and dotted line marks the mean tdTomato intensity in control MNs. (n=3 independent differentiations; bars represent mean ± SD; Mann-Whitney test, individual p-values noted above comparisons).

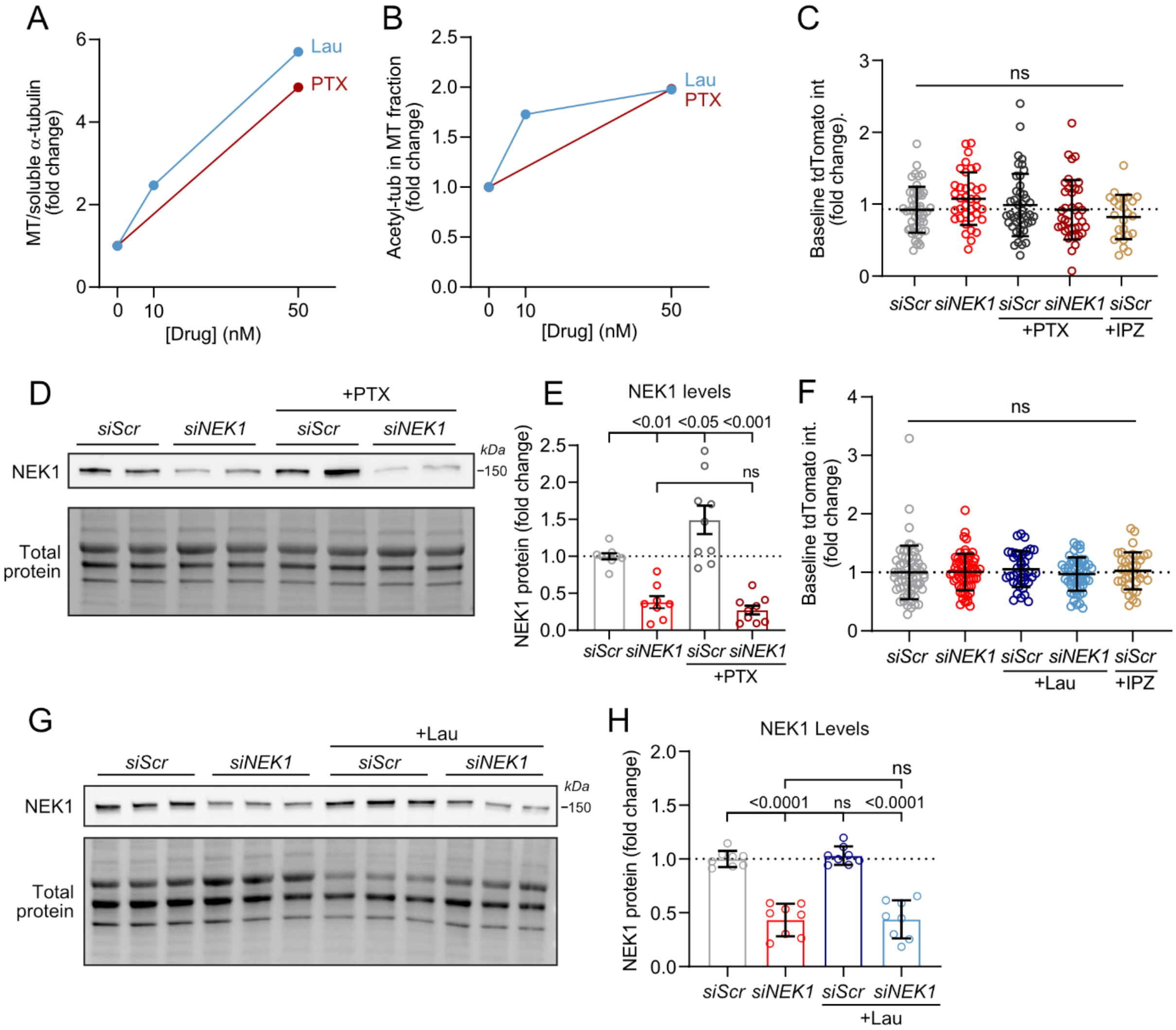
A
MT/soluble α-tubulin (fold change)
[Drug] (nM)
Lau
PTX
B
Acetyl-tub in MT fraction (fold change)
[Drug] (nM)
Lau
PTX
C
Baseline tdTomato int (fold change).
ns
siScr siNEK1 siScr siNEK1 siScr
+PTX +IPZ
D
+PTX
siScr siNEK1 siScr siNEK1
NEK1
kDa
-150
Total protein
E
NEK1 levels
<0.01 <0.05 <0.001
ns
NEK1 protein (fold change)
siScr siNEK1 siScr siNEK1
+PTX
F
Baseline tdTomato int. (fold change)
ns
siScr siNEK1 siScr siNEK1 siScr
+Lau +IPZ
G
+Lau
siScr siNEK1 siScr siNEK1
NEK1
kDa
-150
Total protein
H
NEK1 Levels
ns
<0.0001 ns <0.0001
NEK1 protein (fold change)
siScr siNEK1 siScr siNEK1
+Lau

Fig. S5. Microtubule stabilization restores nuclear import deficits in NEK1-depleted motor neurons.

(**A**) Quantification of relative polymerized:soluble α-tubulin ratio in MN lysates treated with PTX or Lau. Values are based on α-tubulin intensity found within microtubule (MT) and soluble (S) fractions from western blots shown in Fig 5B. (**B**) Quantification of relative acetylated α-tubulin levels within MT fractions of MNs treated with PTX or Lau. Values are based on acetylated α-tubulin intensity found within microtubule (MT) fractions from western blots shown in Fig 5B and normalized to total soluble protein loading. (**C**) Dot plot showing the fold change in whole-cell NES-tdTomato-NLS fluorescence intensity at baseline prior to photobleaching in PTX experiments shown in Fig 5D-F. Individual cells are displayed as circles and dotted line marks the mean tdTomato intensity in control MNs. (n=4 independent differentiations; bars represent mean ± SD; One-way ANOVA, n.s. = not significant, all p-values >0.05). (**D**) Representative western blot showing relative NEK1 protein levels in D40 *siScr*- or *siNEK1*-treated MN lysates following PTX treatment. (**E**) Bar plot showing fold change in NEK1 levels following PTX treatment as described in (D) and normalized to total protein loading. Circles represent individual samples, and the dotted line marks mean NEK1 levels in *siScr*-treated MNs. (n=3 independent differentiations; One-way ANOVA with Tukey's correction; adjusted p-values noted above comparisons). (**F**) Dot plot showing the fold change in whole-cell NES-tdTomato-NLS fluorescence intensity at baseline prior to photobleaching in Lau experiments shown in Fig 5G-I. Individual cells are displayed as circles and dotted line marks the mean tdTomato intensity in control MNs. (n=3 independent differentiations; bars represent mean ± SD; One-way ANOVA, n.s. = not significant, all p-values >0.05). (**G**) Representative western blot showing relative NEK1 protein levels in D40 *siScr*- or *siNEK1*-treated MN lysates following Lau treatment. (**H**) Bar plot showing fold change in NEK1 levels following Lau treatment as described in (G) and normalized to total protein loading. Circles represent individual samples, and the dotted line marks mean NEK1 levels in *siScr*-treated MNs. (n=3 independent differentiations; One-way ANOVA with Tukey's correction; adjusted p-values noted above comparisons).

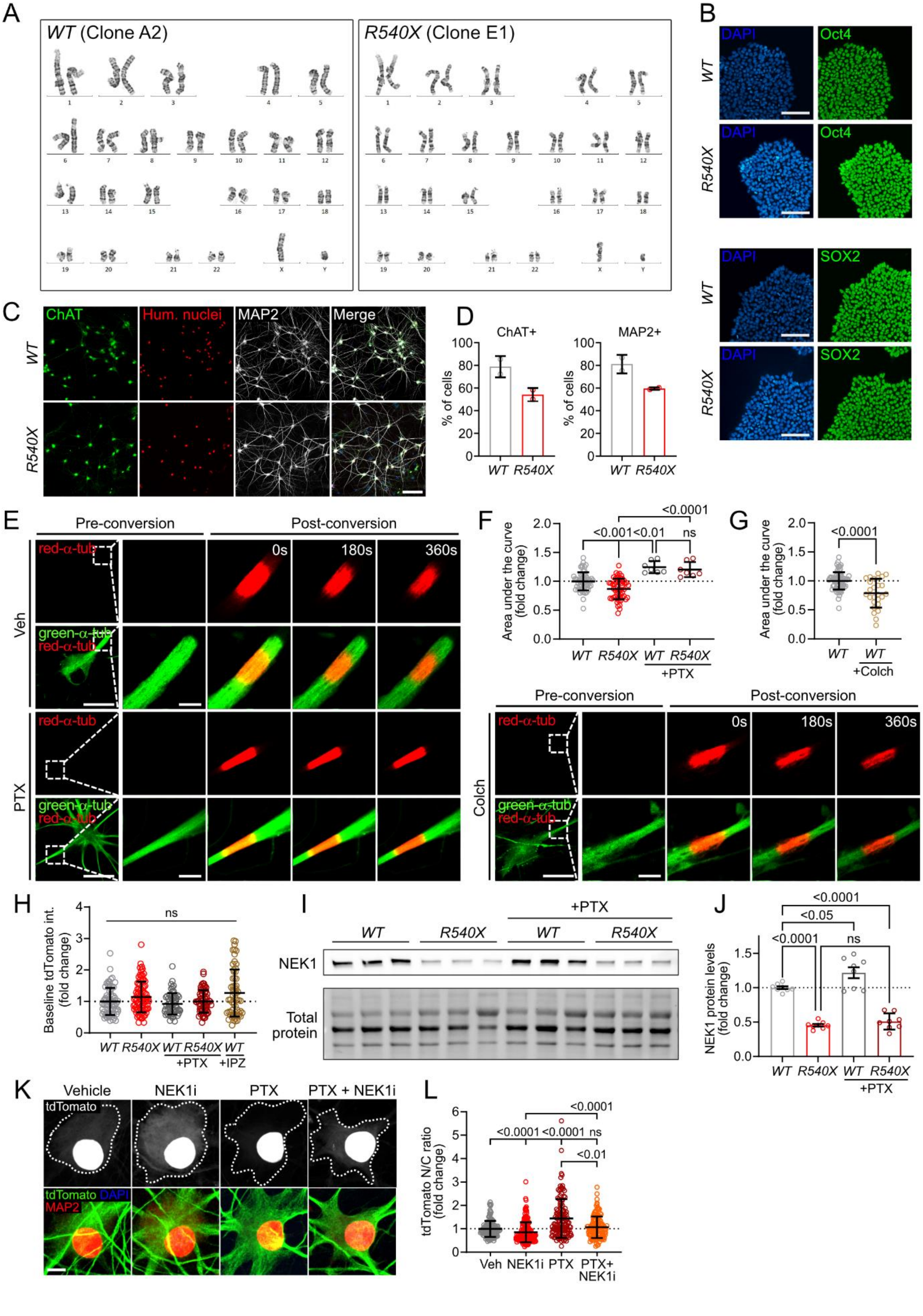

A
WT (Clone A2)
R540X (Clone E1)
B
DAPI
Oct4
SOX2
WT
R540X
C
ChAT
Hum. nuclei
MAP2
Merge
D
ChAT+
MAP2+
% of cells
E
Pre-conversion
Post-conversion
red-α-tub
green-α-tub
0s
180s
360s
Veh
PTX
F
Area under the curve (fold change)
<0.0001
<0.001
<0.01
ns
+PTX
G
+Colch
Colch
H
Baseline tdTomato int. (fold change)
+IPZ
I
NEK1
Total protein
J
NEK1 protein levels (fold change)
<0.05
K
Vehicle
NEK1i
PTX
PTX + NEK1i
tdTomato
DAPI
MAP2
L
tdTomato N/C ratio (fold change)
Veh

Fig. S6. Motor neurons harboring the NEK1-ALS mutation R540X exhibit defects in microtubule homeostasis and N/C transport that can be rescued by PTX.

(**A**) Karyotype analysis of NEK1-WT parental and edited NEK1-R540X iPSC lines. (**B**) Representative confocal images of WT and R540X iPSC colonies following immunostaining for pluripotency markers OCT4 (green, top) and SOX2 (green, bottom). DAPI is shown in blue. Scale bar: 100μm. (**C**) Representative confocal images of D50 WT and R540X MN cultures following immunostaining for ChAT (green), human neuronal nuclei (red), and MAP2 (white). DAPI is shown in blue. Scale bar: 100μm. (**D**) Bar plots showing the percentage of human cells differentiated from WT and R540X iPSCs positive for the MN marker ChAT (left) and neuronal marker MAP2 (right). (n=2 independent differentiations, bars represent mean ± SD). (**E**) Representative confocal images showing tubulin mobility assay in vehicle-, PTX-, and Colch-treated NEK1-WT MN cultures. Dashed lines mark the inset region magnified in the right time-course images. Scale bars: 25μm, 5μm (inset). (**F**) Dot plot of the fold-change in the area under curve (AUC) from tubulin mobility assay shown in (E) in WT and R540X MNs following PTX treatment. Individual cells are displayed as circles and dotted line marks the mean AUC in WT MNs. (n=2 independent differentiations; bars represent mean ± SD, one-way ANOVA with Tukey's correction, adjusted p-values noted above comparisons). (**G**) Dot plot of the fold-change in the area under curve (AUC) from tubulin mobility assay shown in (E) in WT and MNs following Colch treatment. Individual cells are displayed as circles and dotted line marks the mean AUC in WT MNs. (n=3 independent differentiations; bars represent mean ± SD, unpaired t-test, individual p-values noted above comparisons). (**H**) Dot plot showing the fold change in whole-cell NES-tdTomato-NLS fluorescence intensity at baseline prior to photobleaching in experiments shown in Fig 6F-H. Individual cells are displayed as circles and dotted line marks the mean tdTomato intensity in WT MNs. (n=3 independent differentiations; bars represent mean ± SD; Kruskal-Wallis test, n.s. = not significant, all p-values >0.05). (**I**) Representative western blot showing relative NEK1 protein levels in D40 WT and R540X MN lysates following PTX treatment. (**J**) Bar plot showing fold change in NEK1 levels following PTX treatment as described in (I) and normalized to total protein loading. Circles represent individual samples, and the dotted line marks mean NEK1 levels in WT MNs. (n=3 independent differentiations; One-way ANOVA with Tukey's correction; adjusted p-values noted above comparisons). (**K**) Representative images of D50 control MNs expressing the NES-tdTomato-NLS reporter (red) following treatment with vehicle, PTX (50nM), NEK1 inhibitor (1μM), or both PTX and NEK1 inhibitor and immunostaining for MAP2 (green). DAPI is shown in blue. Scale bar = 10μm. (**L**) Dot plot showing the fold-change in NES-tdTomato-NLS reporter N/C ratio following the treatments described in (K). Individual cells are displayed as circles and dotted line marks the mean N/C ratio in vehicle treated MNs. (n=3 independent differentiations; bars represent mean ± SD, Kruskal-Wallis test with Dunn's correction, adjusted p-values noted above comparisons).

iPSC lines utilized in this study								
**Cell line**	**Condition**	**Genetic background**	**Editing**	**Sex**	**Age**	**Reprogramming method**	**Experimental use**	**Source / Reference**
18a	Non-neurological control	18a	None	Female	51	Retrovirus	Figures 1-5, S1-S2, S3A-D, S3G-J, S4C-F, S5	*Boulting et al. (37) RRID: CVCL_8993*
CS002	Non-neurological control	CS0002iCTR-nxx	None	Male	51	Episomal	Figures S3E-F, S4A-B	*Cedars-Sinai/CS iPSC Core Repository; Cat#: CS0002iCTR-nxx*
NEK1-WT	Non-neurological control	KOLF2_C1	None	Male	55-59	Sendai	Figures 6, S6	*Human Induced Pluripotent Stem Cells Initiative (HipSci)*
NEK1-R540X	ALS-linked R540X mutation	KOLF2_C1	R540X mutation introduced	Male	55-59	Sendai	Figures 6, S6	*Human Induced Pluripotent Stem Cells Initiative (HipSci)*
RFP-TUBA1B	Non-neurological control	WTC-11	mTagRFP-T inserted at N-terminus of TUBA1B	Male	30	Episomal	Figures 2B (bottom), S2B	*Coriell/Allen Cell Collection; RRID: CVCL_LK44*

Table S1. iPSC lines utilized in this study.

iPSC source, experimental use and genetic information for the lines used in this study. Patient sex and age at time of sample collection, as well as iPSC reprogramming method, are available in the indicated columns.

TUBA1B						
**Amino acid**	**Sequence**	**NEK1 motif**	**PhosphoSVM score**	**PhosphoSVM rank**	**GPS Score**	**Surface accessibility >0.25?**
**T41**	GQMPSDKtIGGGDDs		0.416	(T) 4	107.69	Yes
**T56**	FNtFFsEtGAGkHVP	[LMFW]-X-X-S/T-[no P]	0.355	(T) 8	n/a	Yes
**T73**	VFVDLEPtVIDEVRt	[LMFW]-X-X-S/T-[no P]	0.154	(T) 21	n/a	Yes
**T80**	tVIDEVRtGtyRQLF	R-1	0.426	(T) 3	n/a	Yes
**T82**	IDEVRtGtyRQLFHP	R/K+2	0.49	(T) 1	2.743	Yes
**T94**	FHPEQLItGKEDAAN	R/K+2	0.376	(T) 5	n/a	Yes
S277	ATyAPVIsAEKAyHE		0.255	(S) 10	2.381	No
**T334**	DVNAAIAtIKtKRsI	R/K+2	0.328	(T) 10	n/a	Yes
**T337**	AAIAtIKtKRsIQFV	R/K+2	0.289	(T) 12	n/a	Yes
**S340**	AtIKtKRsIQFVDWC	R-1	0.395	(S) 5	n/a	Yes
S379	QRAVCMLsNTTAIAE		0.194	(S) 15	2.85	No
**S439**	yEEVGVDsVEGEGEE		0.8	(S) 1	2.43	Yes
**KPNB1**						
**Amino acid**	**Sequence**	**NEK1 motif**	**PhosphoSVM score**	**PhosphoSVM rank**	**GPS Score**	**Surface accessibility >0.25?**
T10	LItILEKtVsPDRLE	[LMFW]-X-X-S/T-[no P]	0.208	(T) 21	n/a	N
**S213**	KANFDKEsERHFIMQ	R/K+2	0.035	(S) 47	n/a	Y
**S526**	GHQNNLRssAyEsLM	R-1	0.183	(S) 14	n/a	Y
**S527**	HQNNLRssAyEsLME		0.315	(S) 6	141.635	Y
S539	LMEIVKNsAkDCYPA	R/K+2	0.123	(S) 26	n/a	N
T587	LQSLLCAtLQNVLRK	[LMFW]-X-X-S/T-[no P]	0.156	(T) 26	n/a	N
S610	ISDVVMAsLLRMFQS		0.07	(S) 37	2.497	N
**S617**	SLLRMFQsTAGsGGV	[LMFW]-X-X-S/T-[no P]	0.111	(S) 29	n/a	Y
**S683**	DLCRALQsNIIPFCD		0.064	(S) 41	2.697	Y
S853	LLTEGRRsKtNKAKT	R-1	0.144	(S) 21	n/a	N
**T855**	TEGRRsKtNKAKTLA	R/K+2	0.25	(T) 15	n/a	Y

Table S2. Predicted NEK1 phosphorylation sites within TUBA1B and KPNB1.

Predicted NEK1 phosphorylation sites within the amino acid sequences of TUBA1B and KPNB1. Phosphorylated amino acids (shown in red font) were identified by PhosphoSite and manually scanned for NEK1 consensus motifs identified in (*43*) which are denoted in the indicated column. PhosphoSVM and GPS 5.0 were then used to predict additional potential NEK1 phosphorylation sites and calculate surface accessibility scores (*40-42*). Bold font within the amino acid column denotes a surface accessibility score of >0.25.

Postmortem patient sample information								
**Patient**	**Patient Code**	**Mutation**	**Sex**	**Age**	**Ethnicity**	**Disease Duration (months)**	**Cause of death**	**PMI (hours)**
**Control #1**	90012	N/A	M	89	W	N/A	Congestive heart failure	16
**Control # 2**	100028	N/A	M	69	W	N/A	Abdominal aortic aneurysm; Hypertension with atherosclerosis	3.25
**NEK1 #1**	80029	**A320E** *NEK1:NM_001199397: exon12:c.C959A*	M	71	W	53	ALS	5
**NEK1 # 2**	120024	**K1052E** *NEK1:NM_001199397: exon31:c.A3154G*	M	71	W	111	ALS	5.5

Table S3. Postmortem patient sample information.

Control and *NEK1*-ALS patient sample information used in Fig. 4D-F, including sex, age, ethnicity, disease duration and cause of death. *NEK1* mutations are shown in the indicated column. Protein changes are reflected in bold text and transcript location of mutations are indicated in italics. PMI = postmortem interval.

Data S1. LC-MS/MS data acquired in siScr- and siNEK1-tread MN cultures.

Differentially expressed proteins in siNEK1-treated motor neurons (shown in Fig. 1D-F, fig. S1F-G), as well as GSEA (shown in Fig. 1G-H) and GO analysis (shown in fig. S1I-J).
